# Risks posed by SARS‐CoV‐2 to North American bats during winter fieldwork

**DOI:** 10.1111/csp2.410

**Published:** 2021-03-30

**Authors:** Jonathan D. Cook, Evan H. C. Grant, Jeremy T. H. Coleman, Jonathan M. Sleeman, Michael C. Runge

**Affiliations:** ^1^ U.S. Geological Survey Patuxent Wildlife Research Center Laurel Maryland USA; ^2^ U.S. Geological Survey Patuxent Wildlife Research Center Turner's Falls Massachusetts USA; ^3^ U.S. Fish and Wildlife Service Hadley Massachusetts USA; ^4^ U.S. Geological Survey National Wildlife Health Center Madison Wisconsin USA

**Keywords:** aerosol transmission, bats, cave surveys, COVID‐19, expert judgment, risk analysis, zoonosis

## Abstract

The virus that causes COVID‐19 likely evolved in a mammalian host, possibly Old‐World bats, before adapting to humans, raising the question of whether reverse zoonotic transmission to bats is possible. Wildlife management agencies in North America are concerned that the activities they authorize could lead to transmission of SARS‐CoV‐2 to bats from humans. A rapid risk assessment conducted in April 2020 suggested that there was a small but significant possibility that SARS‐CoV‐2 could be transmitted from humans to bats during summer fieldwork, absent precautions. Subsequent challenge studies in a laboratory setting have shed new information on these risks, as has more detailed information on human epidemiology and transmission. This inquiry focuses on the risk to bats from winter fieldwork, specifically surveys of winter roosts and handling of bats to test for white‐nose syndrome or other research needs. We use an aerosol transmission model, with parameter estimates both from the literature and from formal expert judgment, to estimate the risk to three species of North American bats, as a function of several factors. We find that risks of transmission are lower than in the previous assessment and are notably affected by chamber volume and local prevalence of COVID‐19. Use of facemasks with high filtration efficiency or a negative COVID‐19 test before field surveys can reduce zoonotic risk by 65 to 88%.

## Graphical abstract

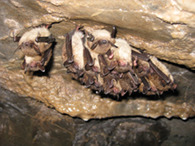

## 1 INTRODUCTION

The novel coronavirus SARS‐CoV‐2 is a generalized pathogen capable of infecting many mammalian species (Gryseels et al., 2020). In humans, SARS‐CoV‐2 infection often results in coronavirus disease 2019 (“COVID‐19”), one of the most widely distributed and deadly diseases in recorded history (Gryseels et al., 2020). Susceptibility to SARS‐CoV‐2 depends on the successful binding of the viral spike protein to host angiotensin‐converting enzyme 2 (ACE2) receptors. Humans are susceptible and proficient transmitters of SARS‐CoV‐2 (Hui et al., 2020), primarily via the respiration of suspended droplets or fine aerosols; transmission from contact with a contaminated surface has limited support (Meyerowitz et al., 2020).

Evidence suggests that SARS‐CoV‐2 evolved in a mammalian host, possibly Old‐World bats in the family *Rhinolophidae* (Zhou et al., 2020). No SARS‐related betacoronaviruses have yet been identified in New‐World bats, but a different type of betacoronavirus has been identified in a New‐World bat from Mexico (Anthony et al., 2013). Because of its origin in Old‐World bats (Zhou et al., 2020) and the potential susceptibility of New‐World bats to the virus, researchers and wildlife managers in North America are concerned about the risks posed by SARS‐CoV‐2 to North American bat populations (Olival et al., 2020).

In April 2020, Runge et al. (2020) conducted an assessment of the risk of SARS‐CoV‐2 infection in bats via contact with humans conducting research, survey, monitoring, wildlife control, and rehabilitation work in summer fieldwork settings. They found that the risk to bats was low if personal protective equipment (PPE) was properly used, but there was significant uncertainty about whether infection could result in spread within bat populations. Information and data have emerged since that risk assessment. This new information includes the results of fruit bat (*Rousettus aegyptiacus*) and big brown bat (*Eptesicus fuscus*) challenge studies (Hall et al., 2020; Schlottau et al., 2020), updates, and better forecasts of human epidemiological data (e.g., human shedding and transmissibility, the importance of aerosols), estimates of the efficacy of different kinds of PPE, information on infection and transmission in other species, and estimates of survival of SARS‐CoV‐2 in different environmental conditions. These new studies can be used to update and expand the assessment of the risks posed by SARS‐CoV‐2 to North American bats.

Natural resource agencies conduct bat population surveys and permit research activities during the winter in North America, when bats congregate in caves, mines, and other hibernacula to hibernate or roost. Congregations can be highly variable in size, from only a handful to many thousands of bats (Kunz, 1982). Winter work involves mainly research, survey, and monitoring (RSM) carried out by State, Tribal, Provincial, and Federal agency personnel and partners, and University faculty, their students, and technicians. This work primarily involves counting individuals in colonies of hibernating bats, including biennial counts of Federally listed species, such as the Indiana bat (*Myotis sodalis*) and Grey bat (*Myotis grisescens*), necessary to assess the status of the species under the U.S. Endangered Species Act (ESA; 16 U.S.C. §1,531 et seq.). Other research activities may involve the handling of bats for investigation for the presence of *Pseudogymnoascus destructans*, white‐nose syndrome (WNS) infection, or to administer and measure the effects of experimental treatments for WNS (e.g., Hoyt et al., 2019). Because winter fieldwork occurs in poorly ventilated cave environments where North American bats are congregating, it is important to evaluate the potential risk of human transmission of SARS‐CoV‐2 to bats resulting from RSM activities. We recognize that the risk of human infection, and thus the risk of transmission to bats during the RSM work, varies geographically and depends on local community incidence of SARS‐CoV‐2.

Managers are faced with multiple competing objectives regarding winter fieldwork. First, if humans introduce SARS‐CoV‐2 to a naïve population of bats, it is possible the novel infection could lead to morbidity and mortality, which may imperil long‐term bat conservation. Further, if a reservoir of SARS‐CoV‐2 becomes established in North American bats, it could represent a source for new exposure and infection in humans; worse, if such a reservoir provides an opportunity for evolution or recombination of the virus, the new viral strains may evade existing immune responses or reduce the efficacy of vaccines or treatments (Huang et al., 2016; Olival et al., 2020; Wang, Wang, & Zhuang, 2020). Second, conservation measures for other threats to bats, including WNS, benefit from ongoing research, and the status of ESA‐listed bat species requires a periodic assessment of population sizes, which occur during winter when many bat species of interest congregate.

Agencies that authorize winter fieldwork on bats have several possible mitigation strategies, including: (a) suspension of work within one or more hibernacula or roost sites; (b) efforts to reduce the risk that field crew is infected with SARS‐CoV‐2; and (c) enhanced PPE (e.g., properly fitted face coverings with high aerosol filtration efficiency) to reduce the potential exposure of bats to the virus. For the suspension of cave work, agencies can issue guidance that no one is to enter a cave for any RSM work for a specified period of time. A milder implementation would involve case‐by‐case analysis of the risk at specific sites, with fieldwork not occurring at sites where the risk was too high. To reduce the risk of field crew shedding virus, individuals could be allowed to enter caves to conduct work only after one, or a combination, of the following mitigation measures are completed: (a) a vaccine is taken, (b) a test for SARS‐CoV‐2 is taken up to 3 days before entering a cave, and that test is negative, or (c) individuals are quarantined for 14 days before entering a cave and show no symptoms. For enhanced PPE, individuals conducting work could be required to wear and maintain functional PPE, including appropriate N95 respirators or other face masks (e.g., cloth masks or polyester surgical masks), eye protection, latex or nitrile gloves, or dedicated clothing (for example, coveralls, Tyvek) to minimize exposure of bats from COVID‐19‐infected individuals.

Estimating the risk to native New World bats is complicated by uncertainties and multiple pathways of exposure, although ongoing research may be used to inform a risk analysis. Experimental infection and transmission trials have been conducted for big brown bats (Hall et al., 2020) and Old‐World fruit bats (Schlottau et al., 2020). While big brown bats did not show evidence of infection or shedding of SARS‐CoV2 (Hall et al., 2020), fruit bats were readily infected and capable of viral‐shedding for a few days postinoculation (Schlottau et al., 2020). Trials of cell tissue cultures of little brown bats (*Myotis lucifugus*) are underway at the USGS National Wildlife Health Center, which will provide evidence for the ability of SARS‐CoV‐2 to infect and replicate in this species.

In this paper, we describe an assessment of the risk of transmission of SARS‐CoV‐2 from human to North American bats during winter fieldwork. We chose three bat species from different genera, with different behaviors and physiology, to represent the bats typically studied in winter. We use a mass balance air circulation model to estimate the probability of aerosol transmission, incorporating empirical estimates of parameters where possible. Where empirical information did not exist, a formal process of expert judgment was used to integrate the best available scientific information, account for uncertainty, and reduce bias in the estimation of key parameters for predictive modeling (Morgan, 2014; Sutherland & Burgman, 2015). As part of this work, we convened an expert panel to estimate a subset of parameters, using a modified Delphi approach with the IDEA (“Investigate, Discuss, Estimate, Aggregate”) protocol (Hanea et al., 2017) and the four‐point elicitation method (Speirs‐Bridge et al., 2010).

## 2 METHODS

### 2.1 Infection risk model

We considered two SARS‐CoV‐2 transmission pathways for North American bats resulting from RSM activities during the winter period, through (a) aerosolized SARS‐CoV‐2 exposure during RSM activities in shared enclosed space without handling; (b) aerosolized SARS‐CoV‐2 exposure during handling; and (c) exposure from SARS‐CoV‐2 deposition on surfaces with subsequent ingestion. The expected number of bats infected with SARS‐CoV‐2, *I*, as a result of a particular survey is given by:

(1) $$E\left[I\right]=E\left[I_{A}+I_{H}\right]=\left(B-H\right)\sigma_{A}\left(1+\gamma_{S}\right)+H\sigma_{H}\left(1+\gamma_{S}\right)$$

where, *B* is the total number of bats in the space being surveyed (i.e., *A + H*); *A* is the number of bats exposed to aerosols, but not handled, during the survey; *H* is the number of bats handled during the survey; *σ*_*A*_ is the combined probability of exposure and infection for an individual bat potentially exposed to aerosols produced by humans (conditional on the specifics of a survey, including the volume of the bat hibernacula or roost, the ventilation rate in the space, the size of the field crew, the probability crew members are shedding virus, and other factors); *σ*_*H*_ is the combined probability of exposure and infection for an individual bat handled by a crew member, under the conditions of a survey; and γ_S_ is a multiplier that captures the additional risk of infection for an individual bat through surface contact with viable virus particles.

The combined exposure and infection probabilities, *σ*_*x*_, are derived parameters that are functions of the characteristics of the enclosed space being surveyed, the crew composition, the prevalence of SARS‐CoV‐2 in the local community, and various mitigation measures taken to reduce risk. The calculation of the exposure and infection probabilities for the two transmission pathways is explained below.

#### 2.1.1 Infection rate in field crew

The human epidemiological parameter of interest is the probability, *p*^+^, that an individual crew member is actively shedding SARS‐CoV‐2 at the time of the survey. We calculate this probability based on two factors: (a) the local prevalence of COVID‐19 in the community in which the crew members live; and (b) adjustments for any mitigation measures the crew takes before the fieldwork, notably voluntary testing, and quarantine before conducting fieldwork.

The probability a crew member is positive can be estimated from county‐level information: the prevalence in their surrounding community (*ψ*), the result of a diagnostic test, and the sensitivity and specificity of the test. Sensitivity (*Sn*) is the probability that an individual with SARS‐CoV‐2 has a positive test, while specificity (*Sp*) is the probability that an individual *who is not infected* has a negative test, and are characteristics of a diagnostic test procedure; different testing methods may have different sensitivity and specificity values. We assume that crew members quarantine before taking a test for SARS‐CoV‐2 (to encompass the latency between exposure and measurable replication), take such a test, receive a negative test result, and quarantine until the survey begins. We are interested in the probability that an individual is infected, given that they receive a negative test for SARS‐CoV‐2. The probability that an individual is infected conditional on a negative test is found using Bayes' Theorem:

(2) $$p^{+}=\frac{\left(1-\mathrm{Sn}\right)\times \psi }{\left(1-\mathrm{Sn}\right)\times \psi +\mathrm{Sp}\times \left(1-\psi \right)}.$$

If a crew member does not take a test, the probability of infection can be estimated as the county‐level prevalence, *ψ*, or by some other method that accounts for the crew member's risk behavior (e.g., https://www.microcovid.org/).

The number of infectious staff, *N*^+^, is a function of the size of the crew, *N*, and the probability that an individual is shedding SARS‐CoV‐2 at the time of the survey, *p*^+^, given by a binomial distribution

(3) $$N^{+}∼\text{binomial}\left(N,p^{+}\right).$$

#### 2.1.2 Pathway 1: Aerosolized SARS‐CoV‐2 exposure in shared enclosed space

The first transmission pathway involves exhaled aerosols from an infected person circulating in the air of an enclosed space and being inhaled by bats. We calculate the transmission risk by considering the amount of aerosol exhaled, the resulting concentration of aerosols in the enclosed space, the respiratory rate of bats, the amount of virus inhaled by bats, and the resulting probability of infection (Figure S1).

We measure exposure in quanta, an indirect, empirically estimated metric based on human epidemiological studies. A quantum is defined as the dose of airborne aerosols required to cause infection in 63% of exposed and susceptible human hosts (Buonanno, Morawska, & Stabile, 2020; Buonanno, Stabile, & Morawska, 2020). The emission rate (in quanta per hour), *E*, of aerosolized SARS‐CoV‐2 by infectious field staff is the product of three components: the hourly per capita quanta emission rate, *Q*_*I*_, a multiplier for the use of PPE, *V*_*PPE*_, and the number of infectious field staff, *N*^+^:

(4) $$E=Q_{I}\times V_{\mathrm{PPE}}\times N^{+}.$$

We assume that the average concentration of aerosolized virus at any location in the cave can be reasonably estimated using a well‐mixed material balance model (Miller et al., 2020), as described in Equation (5). The concentration of infectious aerosols at a given point in time, *C*(*t*), is measured in quanta per cubic meter. Additions to the concentration of aerosols come from emissions by infectious staff, *E* (quanta per hour), which we assume becomes well‐mixed as aerosols throughout the volume, *V*, of the cave. Aerosols are also removed owing to first‐order losses, *λ* (i.e., ventilation, deposition, decay of aerosol). Thus, the change in the concentration of infectious aerosols over time can be expressed as

(5) $$\frac{\mathrm{dC}}{\mathrm{dt}}=\frac{E}{V}-\mathrm{\lambda C}\left(t\right).$$

Solving the differential Equation (5) for *C*(*t*) given the initial condition that *C*(0) = 0 (there is no aerosolized SARS‐CoV‐2 in the cave at the beginning of the survey) gives an equation that describes the buildup of aerosol in the cave during the survey (which has a duration *D*_*E*_)

(6) $$C(t)=\frac{E}{\lambda V}(1‐e^{‐\lambda t}),\mathrm{for}t<D_{E},$$

which has a horizontal asymptote at $\frac{E}{\mathrm{\lambda V}}$.

After staff leave the enclosed space at the end of the survey (at time *D*_*E*_), the concentration of aerosolized virus declines exponentially with loss rate *λ* according to

(7) $$C\left(t\right)=C\left(D_{E}\right)e^{-\lambda \left(t-D_{E}\right)},\text{for}\;t>D_{E}.$$

The total loss rate of aerosols is the sum of three‐loss rates (all in units hr^−1^), owing to ventilation (*λ*_*v*_), the decay of viral particles (*λ*_*DR*_), and deposition of aerosols (*λ*_*DS*_),

(8) $$\lambda =\lambda_{v}+\lambda_{\mathrm{DR}}+\lambda_{\mathrm{DS}}.$$

The ventilation rate, *λ*_*v*_, causes loss of aerosols through airflow through the cave and can be understood as the number of air changes per hour. The deposition rate, *λ*_*DS*_, causes loss as aerosol particles settle to the floor or adhere to other surfaces, thus, no longer circulating. Finally, the decay rate, *λ*_*DR*_, causes loss as viral particles break down and become non‐infectious.

Empirical studies of the viral decay rate for SARS‐CoV‐2 have shown that it is a linear function of environmental conditions (Dabisch et al., 2020),

(9) $$\lambda_{DR}=(0.16030+0.04018\left(\frac{T-20.615}{10.585}\right)\;+0.02176\left(\frac{\mathrm{RH}-45.235}{28.665}\right)+0.14369\left(\frac{S-0.95}{0.95}\right)\;+0.02636\left(\frac{T-20.615}{10.585}\right)\left(\frac{S-0.95}{0.95}\right))\times 60,$$

where *T* is the temperature (studied in the range 10–30°C), *RH* is the relative humidity (20–70%), and *S* is the integrated UVB irradiance (0–1.9 W/m^2^).

**FIGURE 1:**
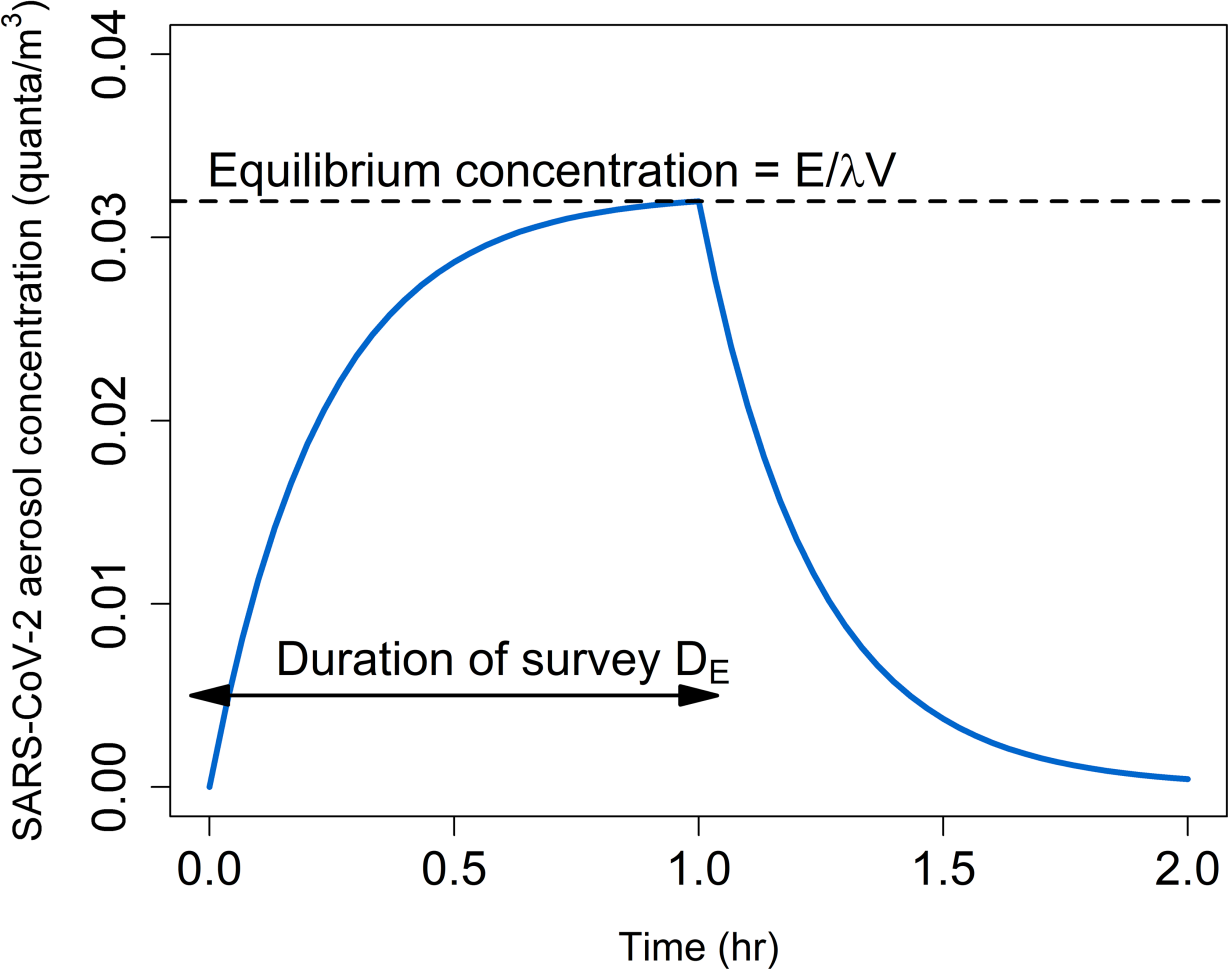
Model of SARS‐CoV‐2 aerosol transmission in an enclosed space over time. The first phase shows the buildup of aerosols during a survey toward an equilibrium level that balances the emission rate against the loss rate. The second phase shows the loss of remaining aerosols through ventilation, decay, and deposition. The graph depicts a scenario in which a single crew member is shedding virus for a 1‐hr survey without wearing a mask in a 500 m^3^ chamber at 10 C and 90% relative humidity, with a ventilation rate of 1.0 hr^−1^

The combined effect of buildup through emission and loss through ventilation, deposition, and decay produces a trajectory of aerosol concentration through time that has two phases: a build‐up toward an asymptotic concentration while the crew is in the enclosed space, and an exponential decline after crew leave the space (Figure 1).

We estimate bat exposure, *Q*_*A*_, as the cumulative amount of aerosolized SARS‐CoV‐2 virus (measured in quanta) inhaled per bat while field staff are active in the cave *and* after field staff depart the cave. The instantaneous exposure (measured in quanta per hour) is the product of the concentration, *C*(*t*), and the species‐specific respiration rate of bats, *R*_*sp*_. The total exposure (measured in quanta per bat) is the integration of this product over time, thus,

(10) $$Q_{A}=\int_{0}^{\infty }R_{\mathrm{sp}}C\left(t\right)\mathrm{dt}$$

$$=R_{\mathrm{sp}}\left[\int_{0}^{D_{E}}\frac{E}{\mathrm{\lambda V}}\left(1-e^{-\mathrm{\lambda t}}\right)\mathrm{dt}+\int_{D_{E}}^{\infty }C\left(D_{E}\right)e^{-\lambda \left(t-D_{E}\right)}\mathrm{dt}\right]$$

$$=R_{\mathrm{sp}}\left[\frac{E}{\mathrm{\lambda V}}\left(D_{E}-\frac{1}{\lambda }\left(1-e^{-\lambda D_{E}}\right)\right)+\frac{E}{\lambda^{2}V}\left(1-e^{-\lambda D_{E}}\right)\right]$$

$$=R_{\mathrm{sp}}\frac{E}{\mathrm{\lambda V}}D_{E}.$$

The probability that a bat is infected, conditional on exposure to aerosolized SARS‐CoV‐2, is a function of two components: the probability that the species is even susceptible to SARS‐CoV‐2, *σ*_*sp*_, and the probability of infection as a function of the dose received. If a species of North American bat is susceptible to SARS‐CoV‐2, the relationship between the probability of bat infection and the magnitude of exposure (i.e., dose of SARS‐CoV‐2 measured in quanta) can be described with a dose–response function. We have defined one quantum as the dose that produces infection in 63% of exposed humans, according to the Wells‐Riley infection model (Miller et al., 2020; Riley, Murphy, & Riley, 1978). We can use the Wells‐Riley infection model to develop a dose–response curve for exposed bats by adding an additional term, *r*, for the sensitivity of bats to the virus, relative to the sensitivity of humans. We assume that all individuals within the same species have the same level of host sensitivity to SARS‐CoV‐2. Thus, the probability of infection as a function of the magnitude of exposure is,

(11) $$\sigma_{A}=\left(1-e^{-r\times Q_{A}}\right)\times \sigma_{\mathrm{sp}}.$$

We also considered a separate route of transmission in Pathway 1 as the probability of infection that may occur when bats are exposed to SARS‐CoV‐2 that has been deposited on cave surfaces (including fur) by an infected individual. Deposition may occur through exhaled droplets or physical contact, and subsequent exposure of bats, through an oral route, that have contact with that surface. Currently, there is no conclusive evidence for direct contact or fomite transmission of SARS‐CoV‐2 in humans (Meyerowitz et al., 2020). Epidemiological evidence strongly suggests that respiratory transmission (i.e., aerosolized virion exposure) is the dominant pathway in human hosts (Lu et al., 2020). Lack of evidence has led some researchers to conclude that exposure to live viruses on surfaces is unlikely to cause secondary infections (Meyerowitz et al., 2020). Nevertheless, natural bat coronaviruses may be primarily transmitted among bats by a fecal‐oral route (Dominguez et al. 2007). Thus, if fecal‐oral transmission is also an important pathway for SARS‐CoV‐2 in bats, surface contact and ingestion through preening could be a plausible mechanism of transmission. We reasoned that these processes (aerosol transmission and surface contact) are intertwined because the source of the virus is the same (exhaled from humans), but we did not understand the surface contact process well enough to develop a mechanistic model. Instead, we indirectly modeled transmission through surface contact as a multiplier to the probability of infection from aerosolized viral exposure. To do so, we estimated the fraction of total infections that occur through surface contact rather than aerosol inhalation, *f*_*S*_, then calculated the additional fraction of bats infected through surface contact as

(12) $$\;\gamma_{S}=\left(\frac{f_{S}}{1-f_{S}}\right),\text{for}\;0\leq f_{S}<1.$$

Taken together, the number of infected bats, *I*_*A*_, from Pathway 1 is a function of the number of bats that are exposed to aerosolized SARS‐CoV‐2, *A*, the probability of infection, *σ*_*A*_, and the multiplier for infection through surface contact, *γ*_*S*_, given by a binomial distribution,

(13) $$I_{A}∼\text{binomial}\left(A,\sigma_{A}\left(1+\gamma_{S}\right)\right).$$

#### 2.1.3 Pathway 2: SARS‐CoV‐2 exposure during handling

The second transmission pathway involves exhaled droplets or aerosols from an infected person being transmitted to a bat during handling. We calculate the transmission risk by considering the amount of aerosol exhaled, the resulting concentration of aerosols in the effective space between the handler and the bat, the respiratory rate of bats, the amount of virus inhaled by bats, and the resulting probability of infection (Figure S1).

The calculations in this pathway largely follow the calculations for Pathway 1, with a few changes. We assume that each bat is only handled by one member of the crew (*n* = 1), so the emission rate of SARS‐CoV‐2 aerosol during the period of handling, *E*_*H*_, has to account for the probability of whether the handler is shedding virus,

(14) $$E_{H}=Q_{I}\times V_{\mathrm{PPE}}\times \text{Bernoulli}\left(\frac{N^{+}}{N}\;\right).$$

Again, we assume a well‐mixed material balance model for aerosol concentration, but we assume the effective volume of the space is the small volume that the handler and the bat share, *V*_*H*_, during the period of handling, *D*_*H*_. Further, we assume that the loss rate is equal to the loss rate of the overall space, *λ*, and that the aerosol exposure can only occur during the period of handling and not thereafter (the aerosolized exposure from Pathway 1 for a handled bat is accounted for below). Thus, the quanta inhaled by the bat during handling is given by,

(15) $$Q_{H}=\int_{0}^{D_{H}}R_{\mathrm{sp}}C\left(t\right)\mathrm{dt}$$

$$=R_{\mathrm{sp}}\int_{0}^{D_{H}}\frac{E_{H}}{\lambda V_{H}}\left(1-e^{-\mathrm{\lambda t}}\right)\mathrm{dt}$$

$$=R_{\mathrm{sp}}\frac{E_{H}}{\lambda V_{H}}\left(D_{H}-\frac{1}{\lambda }\left(1-e^{-\lambda D_{H}}\right)\right).$$

Finally, the probability of infection takes into account both the aerosol exposure during handling, and the aerosol exposure during the full survey (via aerosolized exposure from Pathway 1),

(16) $$\sigma_{H}=\left(1-e^{-r\left(Q_{H}+Q_{A}\right)}\right)\times \sigma_{\mathrm{sp}}.$$

The number of infected bats, *I*_*H*_, from Pathway 2, is a function of the number of bats that are exposed to aerosolized and deposited SARS‐CoV‐2 during handling, *H*, the probability of infection, *σ*_*H*_, and the multiplier for infection through surface contact, *γ*_*S*_, given by a binomial distribution

(17) $$I_{H}∼\text{binomial}\left(H,\sigma_{H}\left(1+\gamma_{S}\right)\right).$$

### 2.2 Parameters estimated from empirical evidence

Thirteen of the 23 parameters in the infection risk model were estimated from published empirical evidence, which was used to develop probability distributions to represent parametric uncertainty (Table 1). The viral emission rate for infected humans (*Q*_*I*_) was estimated from models of respiration (Buonanno, Stabile, & Morawska, 2020,Buonanno, Morawska, & Stabile, 2020) and inference from a documented super spreading event (Miller et al., 2020). The effectiveness of face coverings in reducing emission, including N95 masks, surgical masks, cloth masks, and face shields, was estimated from studies that quantified the filtration efficiencies (Davies et al., 2013; Lindsley et al., 2014; Long et al., 2020). The typical sensitivity and sensitivity of RT‐PCR tests for SARS‐CoV‐2 were estimated from several meta‐analyses of current tests (Arevalo‐Rodriguez et al., 2020; Watson et al., 2020). The ventilation loss rate (*λ*_*v*_) was inferred from several studies of caves (De Freitas et al., 1982; Kowalczk & Froelich, 2010). The loss rate from viral decay (*λ*_DR_) was from laboratory studies that accounted for temperature, relative humidity, and solar irradiation (Dabisch et al., 2020). The loss rate from deposition (*λ*_DS_) was estimated from an empirical study of deposition rates of aerosolized particles (1‐5 μm) that are of comparable diameter to respired aerosolized viral particles (Buonanno, Stabile, & Morawska, 2020; Meyerowitz et al., 2020; Thatcher et al., 2002). Respiration rates for *M. lucifugus* and *E. fuscus* were taken from the literature and were measured at temperatures consistent with hibernacula in winter months (Henshaw, 1968; Hock, 1951; Riedesel & Williams, 1976; Szewczak & Jackson, 1992; Thomas et al., 1990), whereas the respiration rate for *Tadarida brasiliensis* was based on mass‐specific oxygen uptake rates for *E. fuscus* measured at 10 and 20°C and adjusted for differences in body mass (Szewczak & Jackson, 1992). We also derived an elevated respiration rate for *M. lucifugus* with WNS by increasing the healthy bat rate in proportion to increases in evaporative water loss (~53–55% increase) measured by McGuire et al. (2017). Thus, our elevated rate for WNS‐affected *M. lucifugus* considered the increasing waking bouts associated with active infection (Table 1).

**TABLE 1:** Parameters, descriptions, sources, values, and probability distributions used in the infection risk model

Parameter	Definition (unit)	Source	Values (nominal scale; mean ± *SD*)	Probability distributions
Empirical estimates				
*Sp*	Specificity of COVID‐19 PCR test	Arevalo‐Rodriguez et al., 2020; Watson, Whiting, & Brush, 2020	95%	–
*Sn*	Sensitivity of COVID‐19 PCR test	Arevalo‐Rodriguez et al., 2020; Watson et al., 2020	70%	–
Q_I_	Individual viral emission rate. Two levels: Base and active exertion. (quanta/hr)	Buonanno, Stabile, & Morawska, 2020,Buonanno, Morawska, & Stabile, 2020; Miller et al., 2020	Base: 25.1 ± 29.5 (mean ± *SD*); active: 114.4 ± 156.0	truncN(μ = 25.1, σ^2^ = 29.5^2^)^a^; truncN(μ = 114.1, σ2 = 156.0^2^)^a^
*V*_*PPE‐Shield*_	The effectiveness of polycarbonate medical‐grade faceshield (% filtration efficiency)	Lindsley, Noti, Blachere, Szalajda, & Beezhold, 2014	23 ± 3.3	truncN(μ = 23.3, σ^2^ = 3.3^2^)^a^
*V*_*PPE‐Cloth*_	The effectiveness of 2‐ply cloth mask (% filtration efficiency)	Davies et al., 2013	50.9 ± 16.8	truncN(μ = 50.9, σ^2^ = 16.8^2^)^a^
*V*_*PPE‐Surgical*_	The effectiveness of medical‐grade surgical mask (% filtration efficiency)	Davies et al., 2013	89.5 ± 2.7	truncN(μ = 89.5, σ^2^ = 2.7^2^)^a^
*V*_*PPE‐N95*_	The effectiveness of medical‐grade N95 mask (% filtration efficiency)	Long, Woodburn, Berg, Chen, & Scott, 2020	99.4 ± 0.2	truncN(μ = 99.4, σ^2^ = 0.2^2^)^a^
λ_v_	Loss rate from ventilation (% loss/hr)	De Freitas, Littlejohn, Clarkson, & Kristament, 1982; Kowalczk & Froelich, 2010;	Static(s): min = 0.5, max = 1.5; dynamic(d): min = 3, max = 15	s: Unif(min = 0.5, max = 1.5); d: Unif(min = 3, max = 15)
λ_DR_	Loss rate from viral decay (% loss/hr)	Dabisch et al., 2020	Default: 2.20	–
λ_DS_	Loss rate from viral deposition (% loss/hr)	^b^Thatcher, Lai, Moreno‐Jackson, Sextro, & Nazaroff, 2002; Buonanno, Stabile, & Morawska, 2020	Min = 0.2, max = 2.0	Unif(min = 0.2, max = 2.0)
*R*_*SP‐LBB*_	Respiration rate of little brown bat (*Myotis lucifugus*); without and with white‐nose syndrome; (units: m^3^/hr)	Hock, 1951; Henshaw, 1968; Riedesel & Williams, 1976; Thomas, Cloutier, & Gagne, 1990; McGuire, Mayberry, & Willis, 2017	w/o WNS: min = 9.0E−07, max = 6.0E−06; w/WNS: min = 1.4E−06, max = 9.3E−06	w/o WNS: Unif(min = 9.0E−07, max = 6.0E−06); w/WNS: Unif(min = 1.4E−06, max = 9.3E−06)
*R*_*SP‐BBB*_	Respiration rate of big brown bat (*Eptesicus fuscus*); (units: m^3^/hr)	Szewczak & Jackson, 1992	min = 9.1E−06, max = 1.3E−05	Unif(min = 9.1E−06, max = 1.3E−05)
*R*_*SP‐FTB*_	Respiration rate of free‐tailed bat (*Tadarida brasiliensis*); (units: m^3^/hr)	^c^Szewczak & Jackson, 1992	min = 7.5E−06, max = 2.3E−05	Unif(min = 7.5E−06, max = 2.3E−05)
Expert judgment				
*r*	Dose–response multiplier for north American bat species	Expert elicited	0.45 ± 56.86	Group 1: logN(μ = 1.19, σ^2^ = 1.88^2^); group 2: logN(μ = −1.24, σ^2^ = 0.94)
σ _SP‐*LBB*_	Probability that little brown bat (*Myotis lucifugus*) are susceptible to SARS‐CoV‐2; without and with white‐nose syndrome	Expert elicited	w/o WNS: 0.05 ± 0.18; w/WNS: 0.07 ± 0.24	logitN(μ = −3.05, σ^2^ = 1.98^2^)
*σ*_*SP‐BBB*_	Probability that big brown bat (*Eptesicus fuscus*) are susceptible to SARS‐CoV‐2	Expert elicited	0.02 ± 0.12	logitN(μ = −3.83, σ^2^ = 1.86^2^)
*σ*_*SP‐FTB*_	Probability that free‐tailed bat (*Tadarida brasiliensis*) are susceptible to SARS‐CoV‐2	Expert elicited	0.06 ± 0.22	logitN(μ = −2.69, σ^2^ = 2.11^2^)
f_S‐LBB_	Fraction of infections that occur from contact with contaminated surfaces for little brown bat (*Myotis lucifugu*s); without and with white‐nose syndrome	Expert elicited	w/o WNS: 0.25 ± 0.31; w/WNS: 0.24 ± 0.29	w/o WNS: logitN(μ = −2.46, σ^2^ = 3.13^2^); w/WNS: logitN(μ = −2.31, σ^2^ = 2.71)
f_S‐BBB_	Fraction of infections that occur from contact with contaminated surfaces for big brown bat (*Eptesicus fuscus*); without and with white‐nose syndrome	Expert elicited	w/o WNS: 0.19 ± 0.28; w/WNS: 0.22 ± 0.28	w/o: logitN(μ = −2.93, σ^2^ = 2.92^2^); w/WNS: logitN(μ = −2.56, σ^2^ = 2.74)
f_S‐FTB_	Fraction of infections that occur from contact with contaminated surfaces for free‐tailed bat (*Tadarida brasiliensis*)	Expert elicited	0.27 ± 0.33	logitN(μ = −2.23, σ^2^ = 3.19^2^)
Control variables				
*ψ*	Local prevalence of SARS‐CoV‐2	Control variable	Default: 0.05	–
*N*	Number of people in survey crew	Control variable	Default: 5	–
V	Volume of chamber	Control variable	Default: 500 m^3^	‐
V_H_	Effective volume of space between handler and bat	Control variable	Default: 0.76 m^3^	–
D_E_	Duration of survey (hr)	Control variable	Default: 1 hr	–
D_H_	Handling time for an individual bat (hr)	Control variable	Default: 5 min	–
T	Temperature (°C)	Control variable	Default: 10°C	–
RH	Relative humidity	Control variable	Default: 90%	–
S	Solar irradiance	Control variable	Default: 0 W/m^2^	–

*Note*: Parametric uncertainty was included in the model by drawing from probability distributions: truncN, truncated normal distribution (bounded using a minimum of 0).

Abbreviations: logN, lognormal distribution; logitN, logit‐normal distribution; Unif, uniform distribution.

^a^ Truncated normal distributions were bounded using a minimum = 0 and maximum = ***∞*.**

^b^ The loss rate from deposition (*λ*_DS_) was estimated from empirical study of deposition rates of aerosolized particles (1–5 μm) that are of comparable diameter to respired aerosolized viral particles (Buonanno, Stabile, & Morawska, 2020; Meyerowitz et al., 2020; Thatcher et al., 2002).

^c^ The respiration rate for *Tadarida brasiliensis* was based on mass‐specific oxygen uptake rates for *Eptesicus fuscus* measured at 10 and 20°C and adjusted for differences in body mass (Szewczak & Jackson, 1992).

### 2.3 Parameters estimated through expert judgment

Ten of the parameters in the infection risk model were estimated through a formal process of expert judgment (Table 1, Figures S2–S11), using the IDEA protocol (Hanea et al., 2017) with the four‐point elicitation method (Speirs‐Bridge et al., 2010). Twelve experts in virology and bat physiology were recruited (Table S1). Between December 01 and 10, 2020, the experts provided responses to two rounds of questions and participated in three intervening group discussions. The discussions were effective at reducing sources of bias and uncertainty (e.g., linguistic uncertainty) and providing a venue for knowledge‐sharing and conceptual framing. Following the first round of elicitation, we revised several questions and resolved considerable sources of linguistic uncertainty. From round 1 to 2, all 12 experts opted to revise their initial estimates for at least one question; however, considerable epistemic uncertainties remained. We fit probability distributions to the elicited quantiles from each expert independently, by finding the parameters of a logit‐ or log‐normal distribution that minimized the sum of squared differences between the stated and fitted quantiles. For most parameters, a simple linear pool (Clemen & Winkler, 1999) of the individual probability density functions (PDFs) was found (with equal weight across experts), and a probability distribution was fitted to the average PDF by minimizing the Kullback–Leibler distance between a logit‐ or log‐normal distribution and the average PDF (Kullback & Leibler, 1951). However, due to considerable expert uncertainty in the dose–response sensitivity parameter, r, we grouped experts into two groups and fit a mixture distribution that weighted each group according to its size. Thus, in both approaches the aggregate distribution for these parameters encompassed both within‐ and between‐expert epistemic uncertainty about the parameters.

### 2.4 Control variables and infection risk scenarios

The remaining nine variables in the infection risk model were treated as control variables that describe the conditions of individual surveys and were assigned default values typical of winter bat surveys in North America (Table 1). To evaluate infection risk, we developed a set of scenarios intended to represent common conditions under which RSM activities might occur during the winter period. We evaluated the risk of infection to bats as a function of the sampled cave volume (m^3^), the prevalence of COVID‐19 in the localized human community, the number of bats in winter roosts/hibernacula, and the number of bats handled by field staff. We also evaluated the effect of PPE (surgical mask face coverings) and a negative pre‐survey COVID‐19 test as potential mitigation strategies. All risk estimates are summarized as the probability of at least one bat being infected during a survey. Unless specified otherwise, we assumed a staff size of five individuals; a 1‐hr sampling duration; an average bat handling time of 5 min; 1,000 total bats in the hibernacula/winter roost; 25 handled bats in the hibernacula/winter roost; a sampled cave volume of 500 m^3^; cave/roost temperature of 10°C; cave/roost relative humidity of 90%; minimal ventilation in the hibernacula/winter roost (range of λ: 0.5–1.5 hr^−1^); and a local COVID‐19 prevalence of 0.05.

To include the parametric uncertainty in both the elicited and empirical estimates in our infection risk model, we built a stochastic Monte Carlo simulation model in program R. For each parameter, we drew repeated and independent samples from the probability distribution defined for each parameter, as described in Table 1. We then performed the model calculations and quantified the results as the probability that at least one bat was infected during the survey. The Monte Carlo simulation included 5,000,000 replicates.

### 2.5 Sensitivity analysis

To understand the effect of parametric uncertainty on the risk analysis, we conducted a systematic single‐factor sensitivity analysis, examining the maximum difference in the output metric (probability of at least one bat being infected) across the 95% prediction interval (PI) for each parameter. For this analysis, we used the default values for the control variables.

## 3 RESULTS

### 3.1 Species susceptibility

The experts considered a variety of information sources to estimate species susceptibility including, human‐bat ACE2 homology (Damas et al., 2020), the availability of lab‐based challenge studies (Hall et al., 2020), and species ecology. Across individual experts, best estimates for the probability of species susceptibility ranged from 0.01 to 0.25 for *Tadarida brasiliensis*, 0.01–0.20 for *M. lucifugus*, and 0.005–0.10 for *E. fuscus*. In aggregate, the expert panel estimated *Tadarida brasiliensis* to be most likely to be susceptible to SARS‐CoV‐2 (median probability 0.06; 95% PI: 0.001–0.81, Figure S4), followed by *M. lucifugus* (0.05; 95% PI: 0.001–0.70, Figure S2) and *E. fuscus* **(**0.02; 95% PI: 0.001–0.45, Figure S3).

### 3.2 Species susceptibility and Co‐Infection

To evaluate the risk of co‐infection to bats already affected with WNS, experts considered the effect that co‐infection with both WNS and an alpha‐coronavirus had on downregulating the immune response of *M. lucifugus* (Davy et al., 2018). In aggregate, the expert panel estimated that individual bats with active WNS infection may be slightly more susceptible to SARS‐CoV‐2 (0.07; 95% PI: 0.001–0.86, Figure S5) than healthy individuals (0.05; 95% PI: 0.001–0.70, Figure S2).

### 3.3 Dose–Response relationship for SARS‐CoV‐2 in bats

**FIGURE 2:**
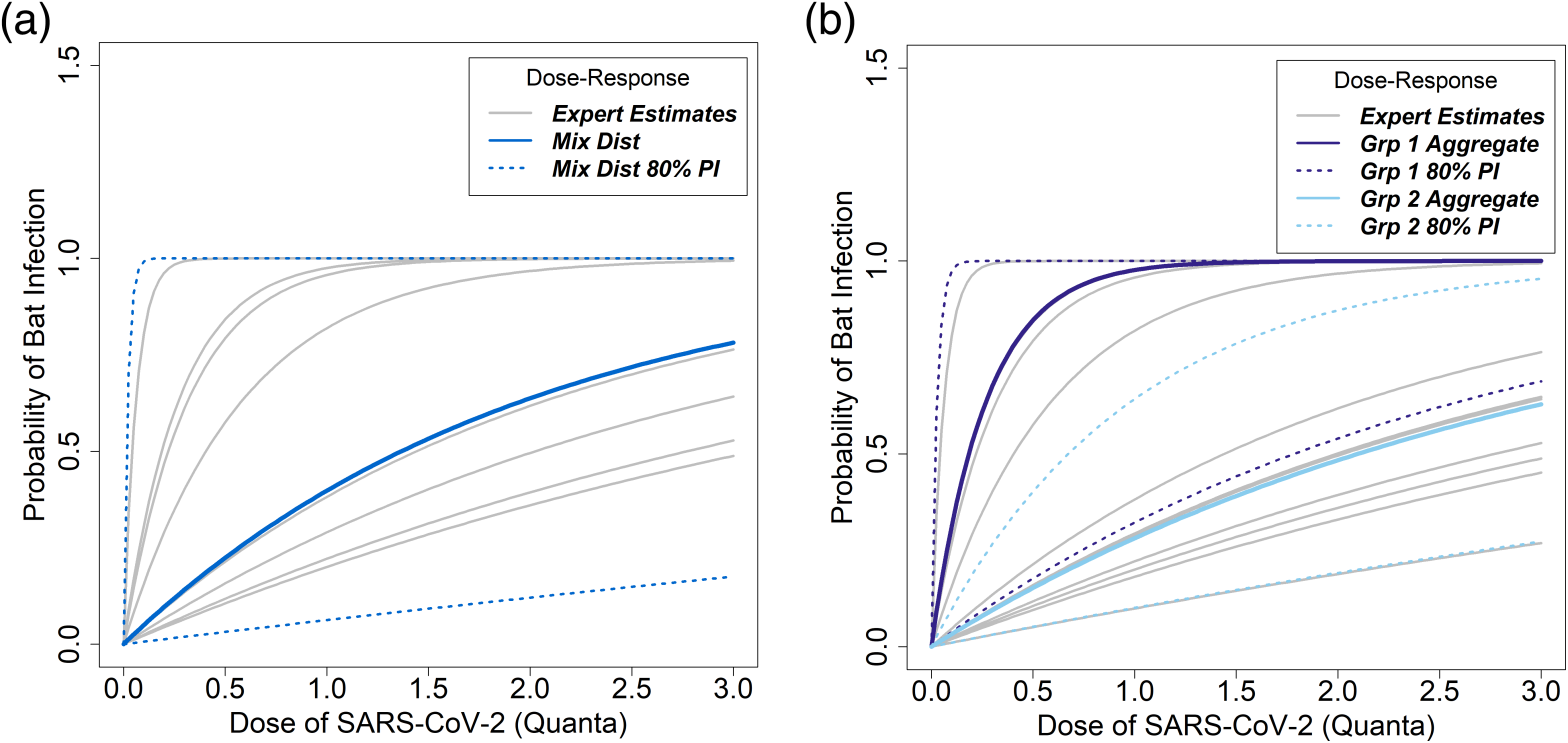
Probability of infection with SARS‐CoV‐2 as a function of dose received by a bat, conditional on susceptibility. Dose is measured in quanta, where one quantum is the viral dose needed to cause infection with probability 0.632 in humans. Gray lines indicate individual expert estimates. (a) The median estimate (bold blue line) from the expert‐weighted mixture distribution and associated 80% confidence interval (dotted blue line) are shown. (b) The aggregate distributions from the two expert groups are shown. Group 1 (purple lines) represents the median and 80% PI of experts who estimated a smaller viral dose was necessary to infect bats relative to humans. Group 2 (light blue lines) represents the median and 80% PI of experts who estimated a larger viral dose was necessary to infect bats relative to humans

In aggregate, the expert panel estimated that, if susceptible to SARS‐CoV‐2, North American bats would require a larger dose of virus to induce infection than humans (Figures 2and S6). Across the 12 experts, the average best estimate of the dose necessary to infect 50% of bats (i.e., ID50) was 3.32 times larger for bats than humans; however, there was also considerable uncertainty amongst experts with best estimates ranging from 0.09 to 9.80 times. Because of the wide range of expert estimates for the bat: human ID50 ratio, the dose–response parameter derived from those data had considerable uncertainty. The group 1 (i.e., experts with median ID50 ratio estimates ≤1, *n* = 4 experts) aggregate estimate for relative dose was 3.28 (95% PI: 0.05–130.87), whereas the group 2 aggregate estimate was 0.29 (95% PI: 0.05–1.83, *n* = 8). Taken together, we combined both groups into a group‐weighted mixture distribution with a median of 0.45 (95% PI: 0.05–49.54). Experts expressed difficulty in estimating the bat: human ID50 ratio because of potential differences in tissue tropism, innate host responses, and the importance of ACE2 receptor homology in bats compared with humans.

### 3.4 Direct transmission from contaminated surfaces

The expert panel evaluated the risk of direct contact transmission relative to aerosol transmission in winter survey settings, with and without the presence of WNS in their hibernacula (Figures S7–S11). Consistent with human epidemiological findings, the experts estimated aerosolized SARS‐CoV‐2 was likely the primary human‐to‐bat transmission route. In aggregate, experts estimated that direct contact risk was highest in *Tadarida brasiliensis* (median probability: 0.10; 95% PI: 0.0002–0.98), and lower in *M. lucifugus* (median probability: 0.08; 95% PI: 0.0001–0.98) and *E. fuscus* (median probability: 0.05; 95% PI: 0.0001–0.94). The primary consideration for the relative risk of direct contact transmission was the elevated activity patterns in *Tadarida brasiliensis* during the winter months. Experts also estimated elevated risk of direct contact transmission to occur in caves with WNS in *M. lucifugus* (median probability: 0.09; 95% PI: 0.0004–0.95) and *E. fuscus* (median probability: 0.07; 95% PI: 0.0003–0.94) as a result of increased activity and co‐infection.

### 3.5 Infection risk

**FIGURE 3:**
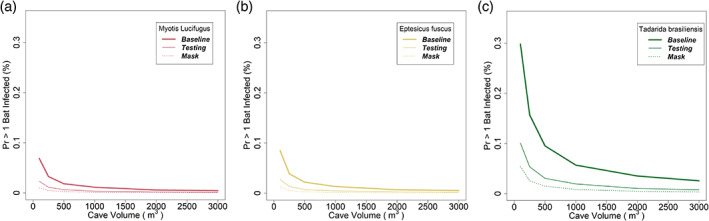
Risk of aerosolized transmission of SARS‐CoV‐2 from humans to bats as a function of cave or roost volume. The dependent axis represents the probability that at least one bat would be infected as a result of a survey, and is expressed in percent, thus, 0.1 represents a probability of 0.001. In each panel, the unmitigated scenario (bold line) is compared with use of pre‐survey testing (thin line) or surgical masks (dashed line) by field staff. (a) *Myotis lucifugus*. (b) *Eptesicus fuscus*. (c) *Tadarida brasiliensis*

**FIGURE 4:**
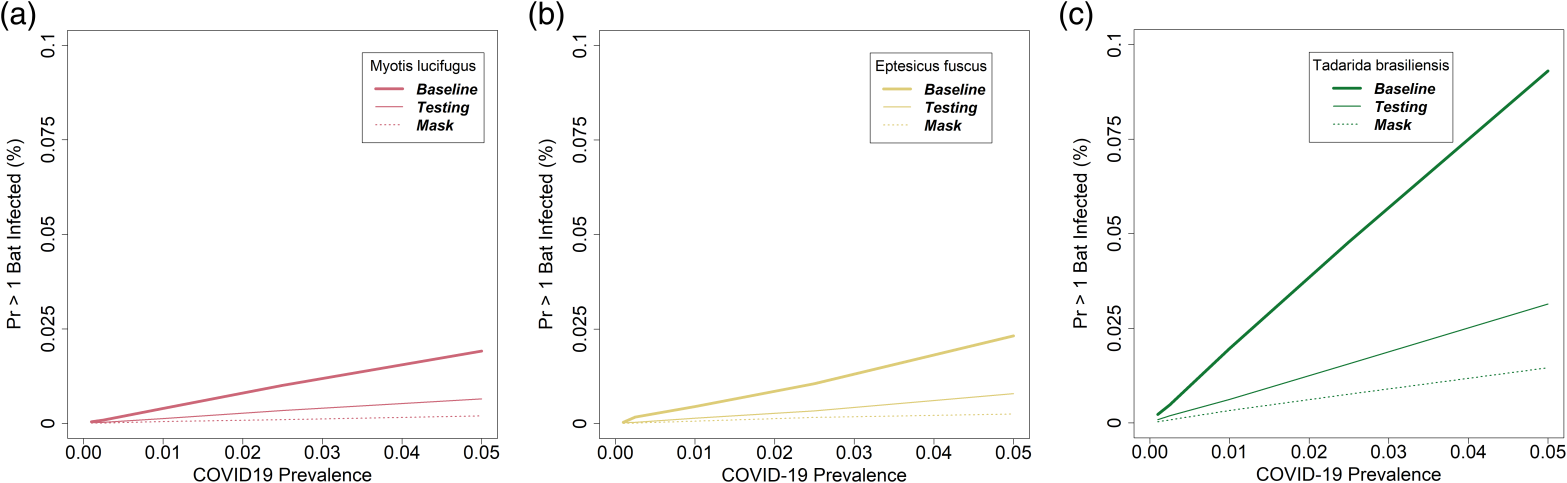
The risk of transmission of SARS‐CoV‐2 from humans to free‐tailed bats (*Tadarida brasiliensis*) as a function of local prevalence of COVID‐19 in humans, in a 500 m^3^ roost with 1,000 bats, 25 of which are handled during the survey. In each panel, the unmitigated scenario (bold line) is compared with use of pre‐survey testing (thin line) or surgical masks (dashed line) by field staff. (a) *Myotis lucifugus*. (b) *Eptesicus fuscus*. (c) *Tadarida brasiliensis*

The results from the infection risk model suggest that the risk of transmission is quite sensitive to the conditions under which a survey is undertaken. Notably, the risk of transmission is driven strongly and inversely by cave volume (Figure 3); for example, the baseline risk of infection of at least 1 free‐tailed bat is 0.095% in a 500 m^3^ chamber, but jumps to 0.298 in a 100 m^3^ chamber (Figure 3c). The risk of transmission is driven linearly by COVID‐19 prevalence in the local human population (Figure 4); for example, the baseline risk of infection rises from 0.001% for free‐tailed bats when the local human prevalence is 0.01 to 0.009% when the prevalence is 0.05 (Figure 4c). Either a negative pre‐survey COVID‐19 test or the use of a surgical mask was estimated to be 65 to 88% effective in reducing the risk of transmission (Figures 3 and 4).

**FIGURE 5:**
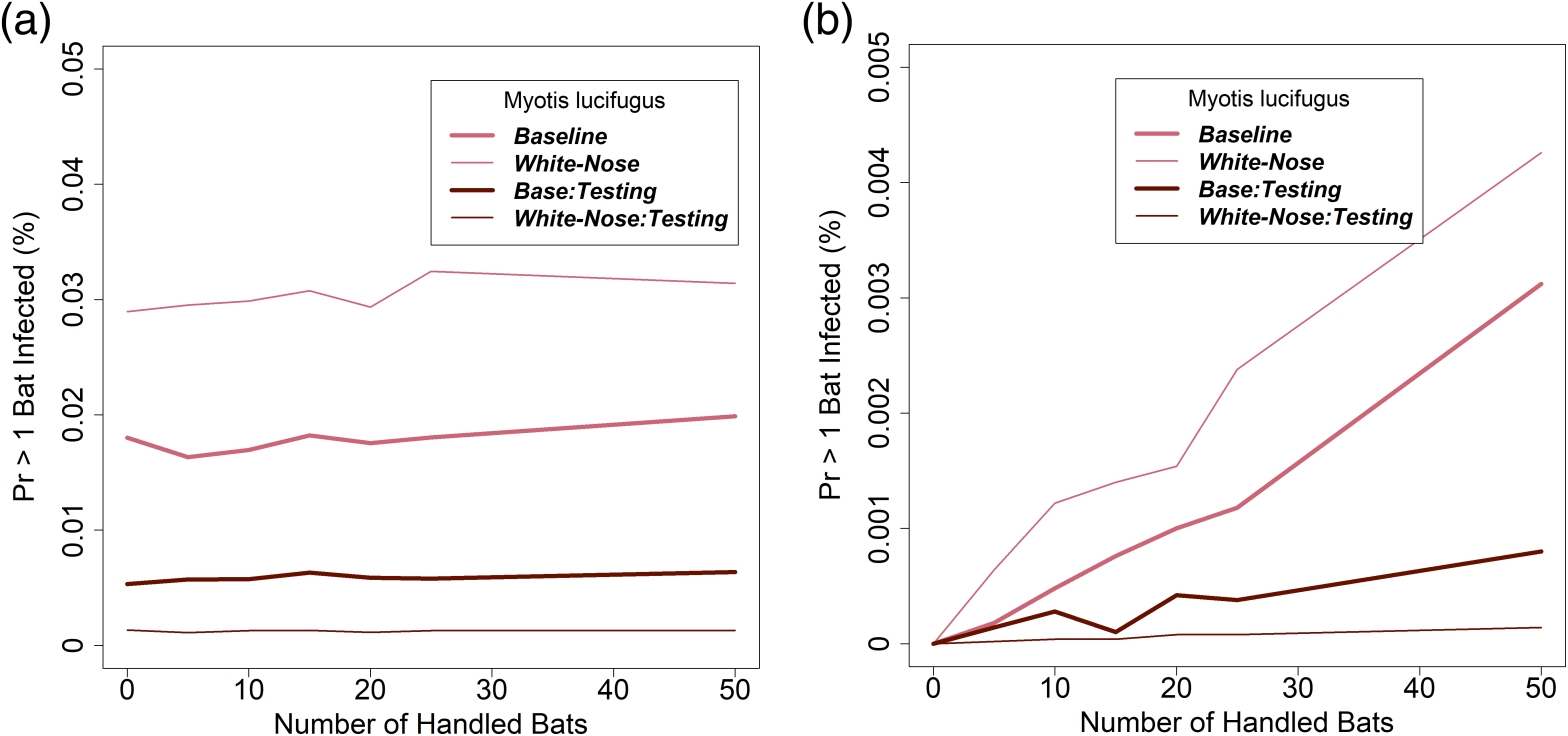
The risk of transmission of SARS‐CoV‐2 from humans to little brown bats (*Myotis lucifugus*) as a function of number of bats handled, with and without the presence of white‐nose syndrome in a 500 m^3^ roost with 1,000 total bats and COVID‐19 prevalence of 0.05. (a)With and without pre‐survey testing of field staff for COVID‐19 and including infection from both Pathways 1 and 2. (b) With and without pre‐survey testing of field staff for COVID‐19 and including infection from Pathway 2 only

The results from the infection risk model suggest that the risk of transmission to bats in caves affected by WNS is approximately twice as high as for corresponding caves without WNS (Figure 5), owing both to increased susceptibility and increased activity (hence, increased respiration rate). Further, across the range of conditions we investigated, the risk of infection was not strongly influenced by the number of bats that were handled (Figure 5), suggesting that most of the transmission is occurring by pathways 1 (aerosol) and 3 (surface contact) rather than pathway 2 (handling).

### 3.6 Sensitivity analysis

The results from the comprehensive sensitivity analysis show that uncertainty in the dose–response parameter for bats (*r*) has the greatest influence on the results (relative sensitivity 1.0), followed by uncertainty in fraction of bat infections from contaminated surface contact (*f*_*S*_; relative sensitivity 0.96), and the probability of susceptibility (σ_sp_; relative sensitivity 0.48) (Table 2). The sensitivity to the remaining parameters is between 3 and 22% of the sensitivity to the dose–response parameter.

**TABLE 2:** Analysis of the sensitivity of the risk of infection to uncertainty in the empirical and expert‐derived parameters

Parameter	Definition (unit)	Source(s)	Uncertainty range	Probability range	Absolute difference	Relative sensitivity
*r*	Dose–response multiplier for bats (three species)	Expert elicited	0.05 – 48.99 (95% prediction interval (PI) upper and lower bounds)	1.18E‐05 – 8.00E‐05	7.99E‐03	1.00
*f*_*s*_	Fraction of bat infections that occur from contact with contaminated surface	Expert elicited	0.0002 – 0.982 (95% PI upper and lower bounds three species)	3.00E‐04 – 8.00E‐03	7.70E‐03	0.96
σ_SP_	Probability of bat susceptibility (three species)	Expert elicited	0.0001 – 0.81 (95% PI upper and lower bounds three species)	8.00E‐07 – 3.80E‐03	3.80E‐03	0.48
Q_I_	Aerosolized viral emission rate (quanta/hr)	Buonanno, Stabile, & Morawska, 2020,Buonanno, Morawska, & Stabile, 2020; Miller et al., 2020	3.4 – 390.5 (95% confidence interval (CI) upper and lower bounds)	3.40E‐05 – 1.80E‐03	1.77E‐03	0.22
*R*_*SP*_	Bat respiration rate (m^3^/hr)	Hock, 1951; Henshaw, 1968; Riedesel & Williams, 1976; Thomas et al., 1990; McGuire et al., 2017	9E‐07 – 1.3E‐05 (minimum and maximum three species)	8.54E‐05 – 9.00E‐04	8.15E‐04	0.10
λ_v_	Loss rate from ventilation (% loss/hr)	De Freitas et al., 1982; Kowalczk & Froelich, 2010	0.5 – 15 (minimum and maximum reported values)	1.00E‐03 – 3.00E‐04	7.00E‐04	0.09
λ_DR_	Loss rate from viral decay (% loss/hr)	Dabisch et al., 2020	0.5 (5°C, 70%RH) – 3.0 (15°C, 99%RH)	1.00E‐03 – 7.00E‐04	3.00E‐04	0.04
λ_DS_	Loss rate from viral deposition (% loss/hr)	Thatcher et al., 2002; Buonanno, Stabile, & Morawska, 2020	0.2 – 2.0 (minimum and maximum reported values)	1.00E‐03 – 8.00E‐04	2.00E‐04	0.03

*Note*: The absolute difference shows the maximum difference in the probability of at least 1 bat being infected during a survey across the range of the parameter being investigated. The relative sensitivity re‐scales the absolute difference in comparison to the maximum absolute difference (for the dose–response multiplier, *r*).

**FIGURE 6:**
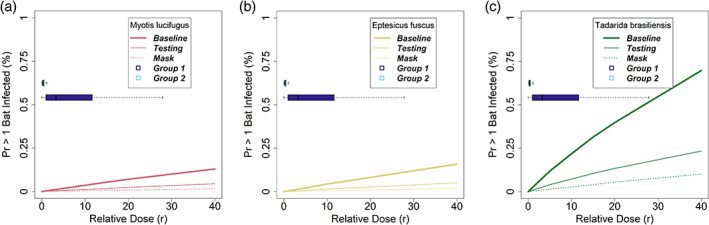
The sensitivity of risk of transmission of SARS‐CoV‐2 from humans to little brown bats (*Myotis lucifugus*) as a function of the relative dose parameter, r. In each panel, the unmitigated scenario (bold line) is compared with use of pre‐survey testing (thin line) or surgical masks (dashed line) by field staff. The boxplots indicate the range of uncertainty for the two expert groups for the dose parameter. (a) *Myotis lucifugus*. (b) *Eptesicus fuscus*. (c) *Tadarida brasiliensis*

The results of the infection risk model were highly sensitive to uncertainty in the dose–response relationship (Figure 6). During the expert elicitation, the experts expressed the most concern about this parameter, because it could be affected by many factors (tissue tropism, innate immune response of bats, ACE2 homology, adaptation of the virus to human systems), none of which are well understood yet. Over the wide range of uncertainty expressed by the experts about the infectious dose for bats relative to humans, the risk of infection varies widely (Figure 6).

## 4 DISCUSSION

Our analysis of the risk of transmission of SARS‐CoV‐2 to bats, resulting from winter fieldwork in enclosed spaces where bats are hibernating or roosting, finds that there is a small but non‐negligible risk if no protective measures are employed. We adapted an aerosol model, developed for understanding the transmission of SARS‐CoV‐2 among humans (Miller et al., 2020), to represent conditions during winter field surveys. By dividing infection risk into two pathways that included exposure risk from aerosolized virus and virus‐contaminated surfaces, we could consider control measures that we expected would reduce the risk of exposure, and that could be implemented by management agencies, university researchers, and their personnel when conducting winter fieldwork. We found that these control measures markedly reduced risk.

The level of risk depends on factors specific to each cave, mine, or roost site. Because the exposure to SARS‐CoV‐2 depends on aerosol transmission, characteristics of airflow, the number of bats, and the size of the surveyed areas influence the risk, as does the prevalence of COVID‐19 in the local human population. Sites, where bats congregate, are highly variable, where some locations require close proximity of field staff to bats, in confined spaces with little airflow. The risk of exposure and transmission in these locations is elevated relative to large spaces where aerosols are diluted in the larger volume and have a smaller chance of infecting torpid bats on the hibernacula ceilings.

For critical fieldwork that aids in the conservation of bat populations, managers have several options to reduce the risk of transmitting an infection to bats. Suspension of fieldwork may be warranted on a case‐specific basis. Based on our results, large sites with small, expected population sizes, in areas of the United States with low COVID‐19 prevalence in the human population result in the lowest risk of bat infection. Field personnel that test negative, have received a full course of a vaccine, or complete a 14‐day quarantine without symptoms reduce the probability of infection; however, because of the moderate sensitivity of current COVID‐19 tests (~70%; Arevalo‐Rodriguez et al., 2020; Watson et al., 2020), less than 100% immunity from current COVID‐19 vaccines (e.g., Polack et al., 2020), and potential for exposure even while in quarantine, the risk of infected staff in communities with elevated COVID‐19 prevalence is not zero. Further, our findings suggest that bats that are in close proximity to humans (e.g., for swabbing for WNS, or to assess body condition during hibernation) are likely at elevated risk of disease exposure and are thus, more likely to become infected. We find that the use of PPE (properly fit cloth or surgical facemasks, or N95 respirators) reduces risk substantially, and we assume that individuals are wearing masks that retain effectiveness during the duration of a survey. For N95 masks, effectiveness will require fit testing and training on proper use. Because of the high‐humidity environment of typical surveys, masks will be more effective if they are replaced regularly before they become saturated (Dbouk & Drikakis, 2020).

In an earlier risk assessment focused on spring and summer fieldwork conditions, Runge et al. (2020) had to rely almost entirely on expert judgment for parameters in their risk model. Since that time, a substantial amount of empirical data have accrued, and the risk assessment described here was able to incorporate empirical estimates for over half of the parameters. Nevertheless, there remained several key parameters in our model for which empirical information does not yet exist. Like Runge et al. (2020), we used a formal process of expert elicitation to obtain estimates of unknown parameters. Expert elicitation can result in reliable predictions (e.g., Adams‐Hosking et al., 2016; Martin et al., 2012; O'Hagan et al., 2006; Runge, Converse, & Lyons, 2011; Speirs‐Bridge et al., 2010). Importantly, the values provided by experts are not substitutes for empirically derived parameters; rather they allow an estimate of risk, with associated uncertainty, to be used until empirical estimates are available.

Despite the accrual of substantial empirical results to inform disease transmission pathways and previously unknown parameters, there remains considerable uncertainty in human‐to‐bat SARS‐CoV‐2 transmission risk. For instance, the dominance of aerosolized transmission of SARS‐CoV‐2 among humans may not be entirely translatable to transmission dynamics from humans‐to‐bats. Other known bat coronaviruses appear to be adapted to fecal‐oral routes of transmission as evidenced by higher viral shedding in feces relative to respiratory secretions (Dominguez et al. 2007). Thus, it remains unknown how important aerosolized respiratory transmission may be in North American bat species. Further, many of the mechanisms of viral binding and replication remain areas of active epidemiological study. As a result, the effective dose necessary to initiate infection in wild species is challenging to estimate; however, in other disease systems (e.g., rabies) smaller wildlife species can require higher vaccination doses to trigger an immune response which is consistent with our estimate of a higher dose necessary to initiate infection in bats relative to humans (Te Kamp et al., 2020).

The specific details of the COVID‐19 pandemic continue to change quickly. This analysis, including the expert elicitation, were conducted in early December 2020, before awareness of more transmissible variants (like the UK variant B.1.1.7) was common. Thus, this analysis does not account for the possibility that the properties of the circulating virus may change, nor did the experts consider whether new variants would differ in their potential effect on bats.

A challenge in making management decisions is how humans deal with uncertainty. Uncertainty is an inability to precisely know something. In making forecasts, uncertainty may be expressed as a probability of expecting each of a range of possible outcomes; some of those outcomes will be better than others. A risk analysis is composed of four interrelated parts (OIE & IUCN, 2014). First is the identification of a hazard (like a pathogen) that might damage a resource of value (e.g., bat populations). The second step provides decision‐makers with an objective and defensible method of assessing the risk associated with the hazard of an undesired outcome for an objective of interest. Our model was constructed to provide this objective estimate of risk for RSM work conducted by State, Tribal, Provincial, and Federal agencies in support of bat conservation objectives. The third step evaluates the management options and their ability to mitigate the risk of negative outcomes. In our application, the evaluation was based on the desire of agencies to use enhanced PPE and COVID‐19 testing to minimize zoonotic spillover of SARS‐CoV‐2 from humans to bats. The last step is the implementation of management actions to mitigate the identified risk. An assessment of whether and how risk should be mitigated will depend on the values and risk tolerances of the decision‐makers and stakeholders. Importantly, the conservation of bat populations and the management of their hibernacula and roost sites often involves other objectives unrelated to bats. Mitigation decisions may involve tradeoffs among these competing objectives, which agencies may assess using formal decision analysis.

Ultimately, how agencies use this decision framing and risk assessment may differ across agencies, taking into account their specific mandates. Different decision contexts may tolerate varying amounts of risk (i.e., agencies may have different acceptable levels of protection), and thus, may choose to implement different sets of mitigation actions. For our results we chose to examine mitigation options separately, but in practice, our model allows for the evaluation of integrated mitigation elements, such as the combination of masks and testing. Our analysis shows that bats are at risk of SARS‐CoV‐2 infection from humans during winter surveys, but mitigation may reduce the risk to satisfactory levels, allowing conservation research to continue in some cases.

## CONFLICT OF INTEREST

The authors declare no conflicts of interest.

## AUTHORS CONTRIBUTIONS

Jonathan D. Cook, Evan H. Campbell Grant, and Michael C. Runge conceived the ideas and designed methodology; Jonathan D. Cook, Evan H. Campbell Grant, and Michael C. Runge collected the data; Jonathan D. Cook and Michael C. Runge analyzed the data; Jonathan D. Cook, Evan H. Campbell Grant, Jeremy T. H. Coleman, Jonathan M. Sleeman, and Michael C. Runge led the writing of the manuscript. All authors provided important contributions to drafts and gave final approval for publication.

## ETHICS STATEMENT

We assert that this research is new and has not been reviewed, or published, elsewhere. All authors have read and approved the final version of the submitted manuscript and have made substantive contributions to this work.

## Supporting information


**Appendix** S1: Supporting information

## Data Availability

The data underlying this article will be shared on reasonable request to the corresponding author.
