# Supplementary material for: Risks posed by SARS‐CoV‐2 to North American bats during winter fieldwork
Source: Conserv Sci Pract. 2021 Mar 30;3(6):e410. doi: 10.1111/csp2.410 (PMC8250205; doi:10.1111/csp2.410)

**Supporting Information**

**Risks Posed by SARS-CoV-2 to North American Bats during Winter Field Work**

Supporting Tables and Figures.

**Table S1.** Names and affiliations for the 12 expert panel participants.

| Expert Name | Affiliation |
| --- | --- |
| Dr. Kaitlin Sawatzki | Tufts University |
| Dr. Nathan Fuller | Texas Parks and Wildlife |
| Dr. Amy Gilbert | U.S. Department of Agriculture |
| Mr. Cory Holliday | The Nature Conservancy |
| Dr. DeeAnn Reeder | Bucknell University |
| Dr. Tony Schountz | Colorado State University |
| Dr. Winifred Frick | University of California - Santa Cruz |
| Mr. Theodore Weller | U.S. Department of Agriculture |
| Dr. Allen Kurta | Eastern Michigan University |
| Mr. Daniel Neubaum | Colorado Parks and Wildlife |
| Dr. Jonathan Towner | Center for Disease Control and Prevention |
| Dr. Michelle Verant | National Park Service |

**Figure S1.** Conceptual diagram of infection risk model including two transmission pathways. Pathway 1 is a combination of two transmission routes: aerosolized SARS-CoV-2 exposure and probability of infection for bats in a shared enclosed space and the probability of infection from contact with a SARS-CoV-2 contaminated surface. Pathway 2 is a combination of two transmission routes: aerosolized SARS-CoV-2 exposure and probability of infection for bats that are handled during RSM activities and the probability of infection from contact with a SARS-CoV-2 contaminated surface. The numeric parenthetical references correspond to the numbered equations in the body of the manuscript.


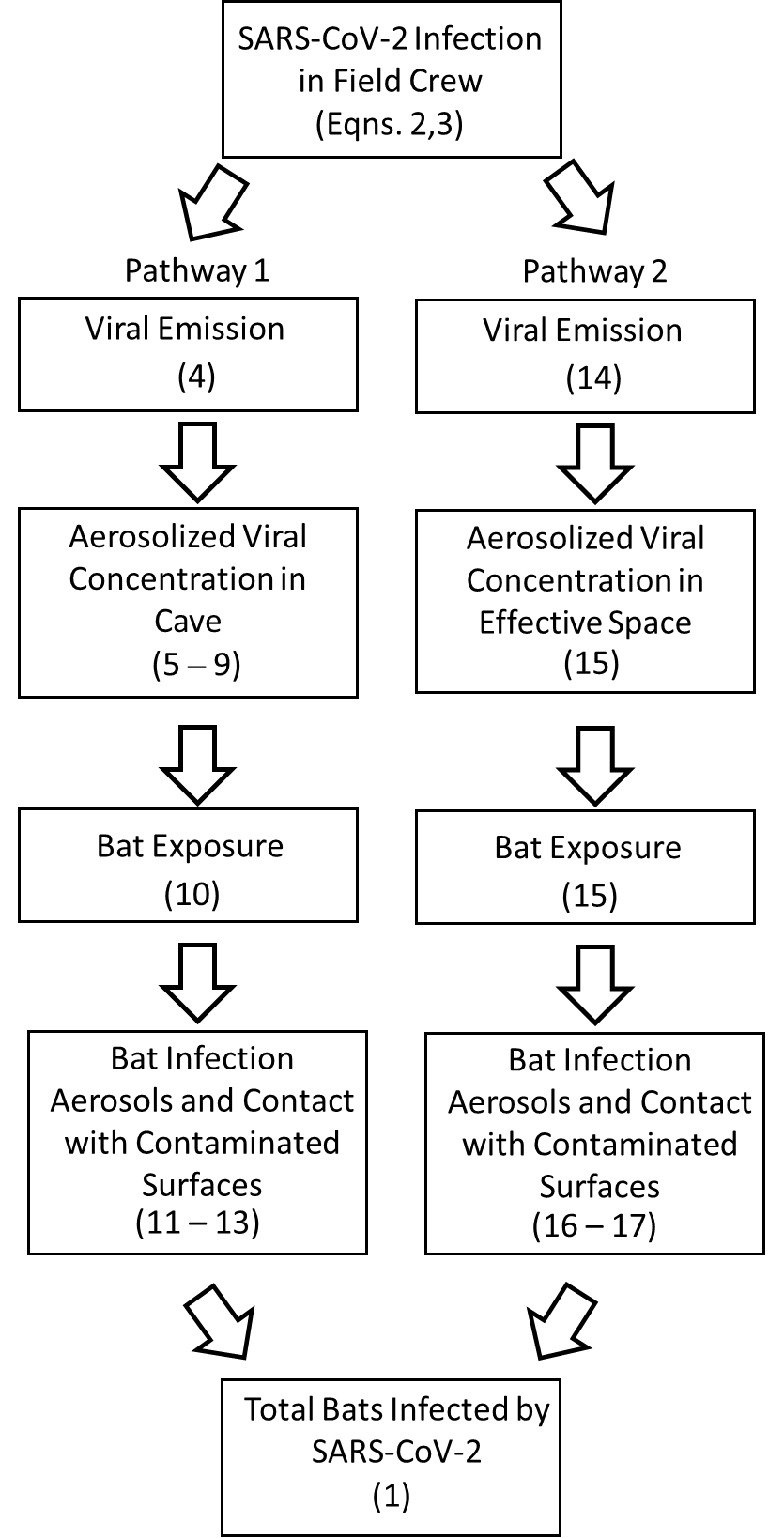


**Figure S2.** Expert panel responses to Question 1 — What is the probability that little brown bats (*Myotis lucifugus*) are susceptible to SARS-CoV-2? (A) fitted probability distributions for individual experts, and (B) average and fitted distributions across experts. The aggregate distribution has a median of 0.05 and an 80-percent confidence interval of (0.003, 0.373).


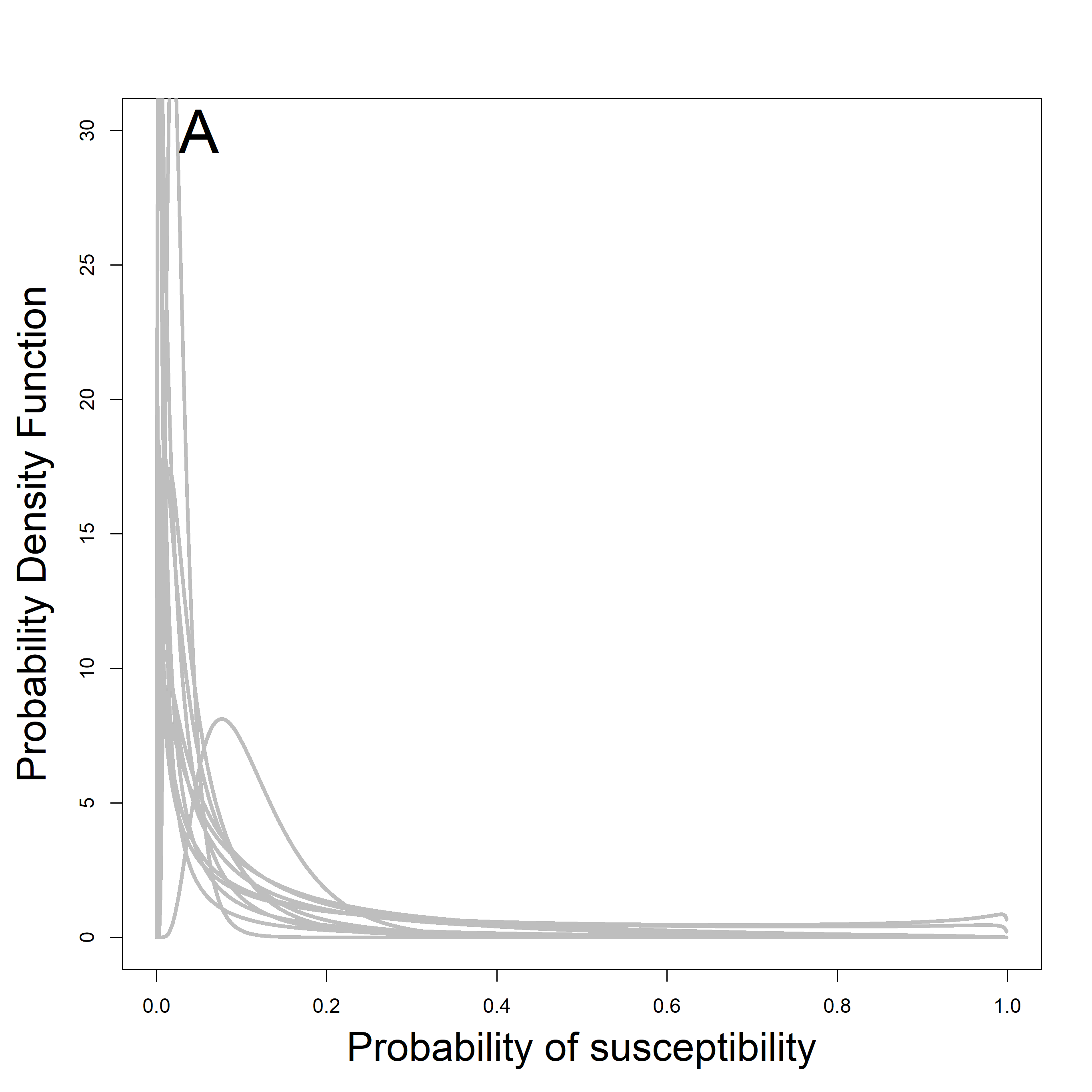

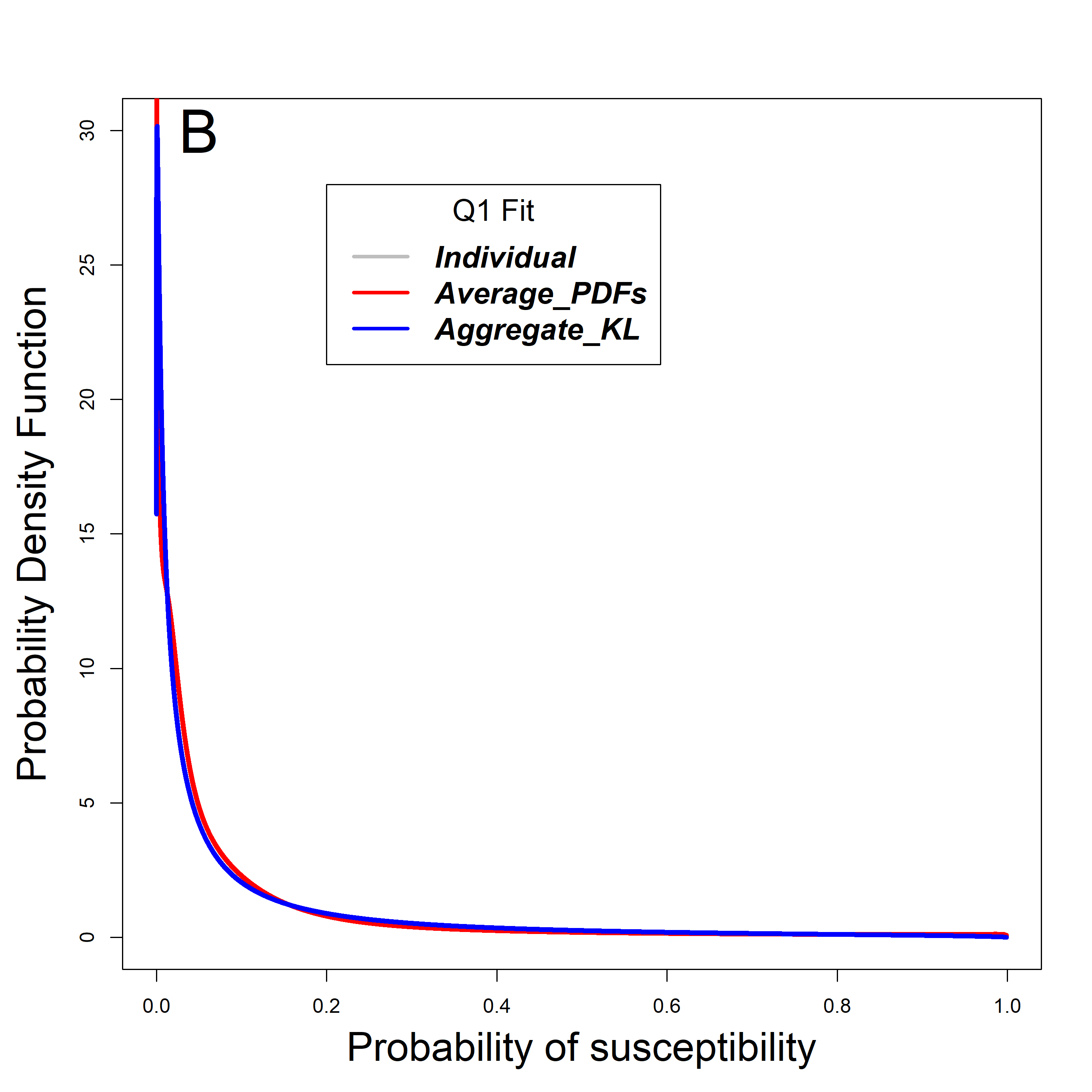


**Figure S3.** Expert panel responses to Question 2 — What is the probability that big brown bats (*Eptesicus fuscus*) are susceptible to SARS-CoV-2? (A) fitted probability distributions for individual experts, and (B) average and fitted distributions across experts. The aggregate distribution has a median of 0.02 and an 80-percent confidence interval of (0.002, 0.189).


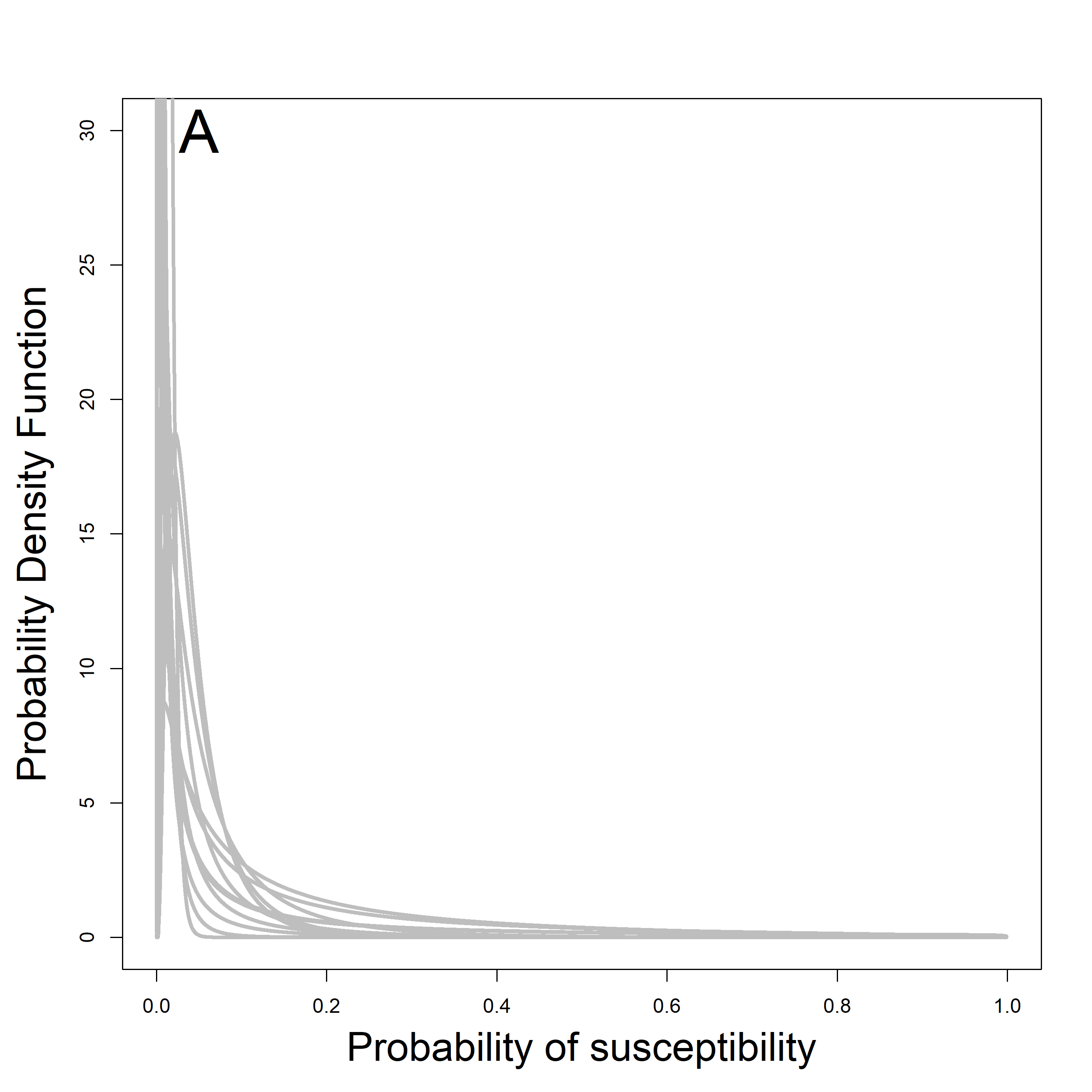

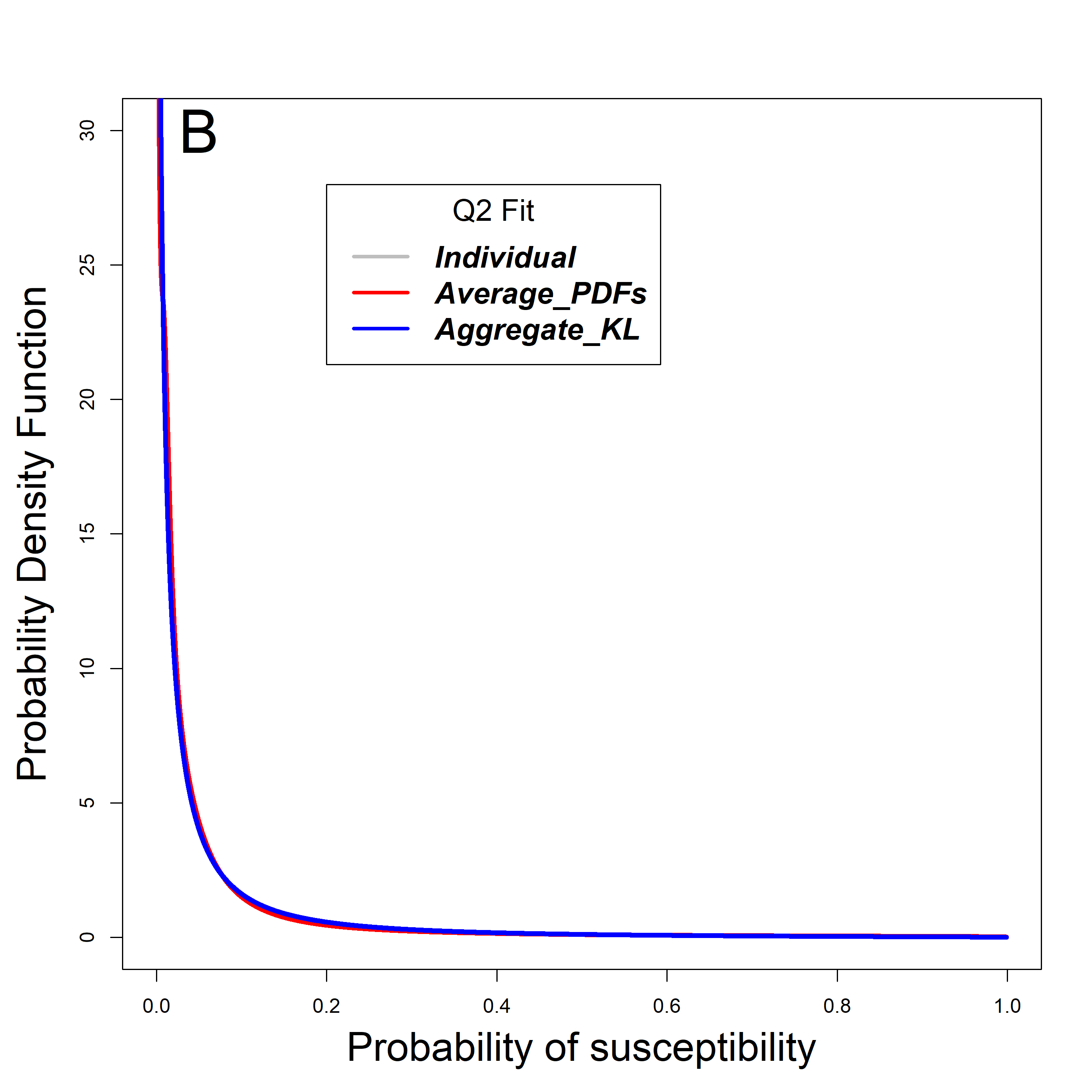


**Figure S4.** Expert panel responses to Question 3 — What is the probability that free-tailed bats (*Tadarida brasiliensis*) are susceptible to SARS-CoV-2? (A) fitted probability distributions for individual experts, and (B) average and fitted distributions across experts. The aggregate distribution has a median of 0.06 and an 80-percent confidence interval of (0.004, 0.5).


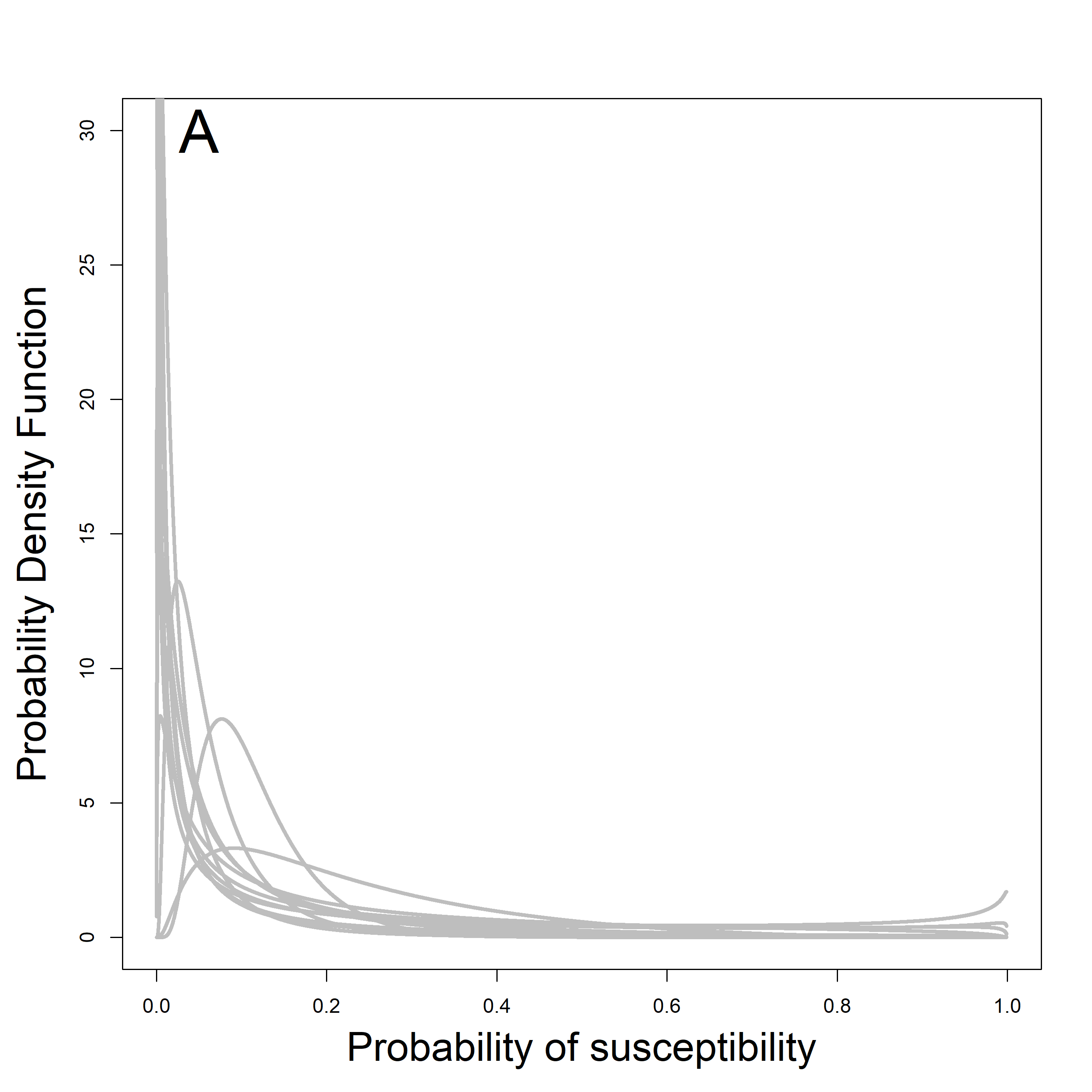

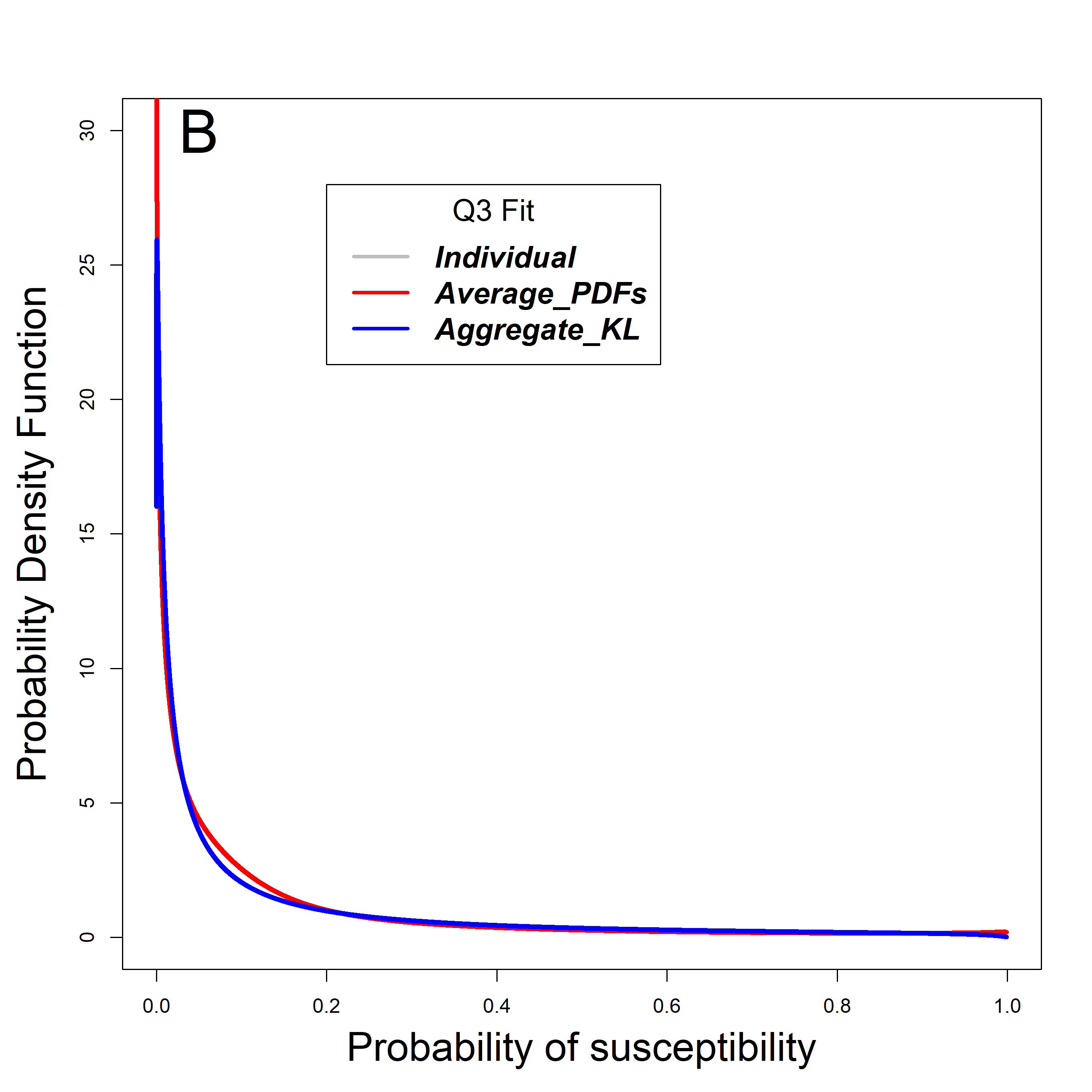


**Figure S5.** Expert panel responses to Question 4 — What is the probability that little brown bats (*Myotis lucifugus*) that are currently infected with white-nose syndrome are susceptible? (A) fitted probability distributions for individual experts, and (B) average and fitted distributions across experts. The aggregate distribution has a median of 0.07 and an 80-percent confidence interval of (0.004, 0.58).


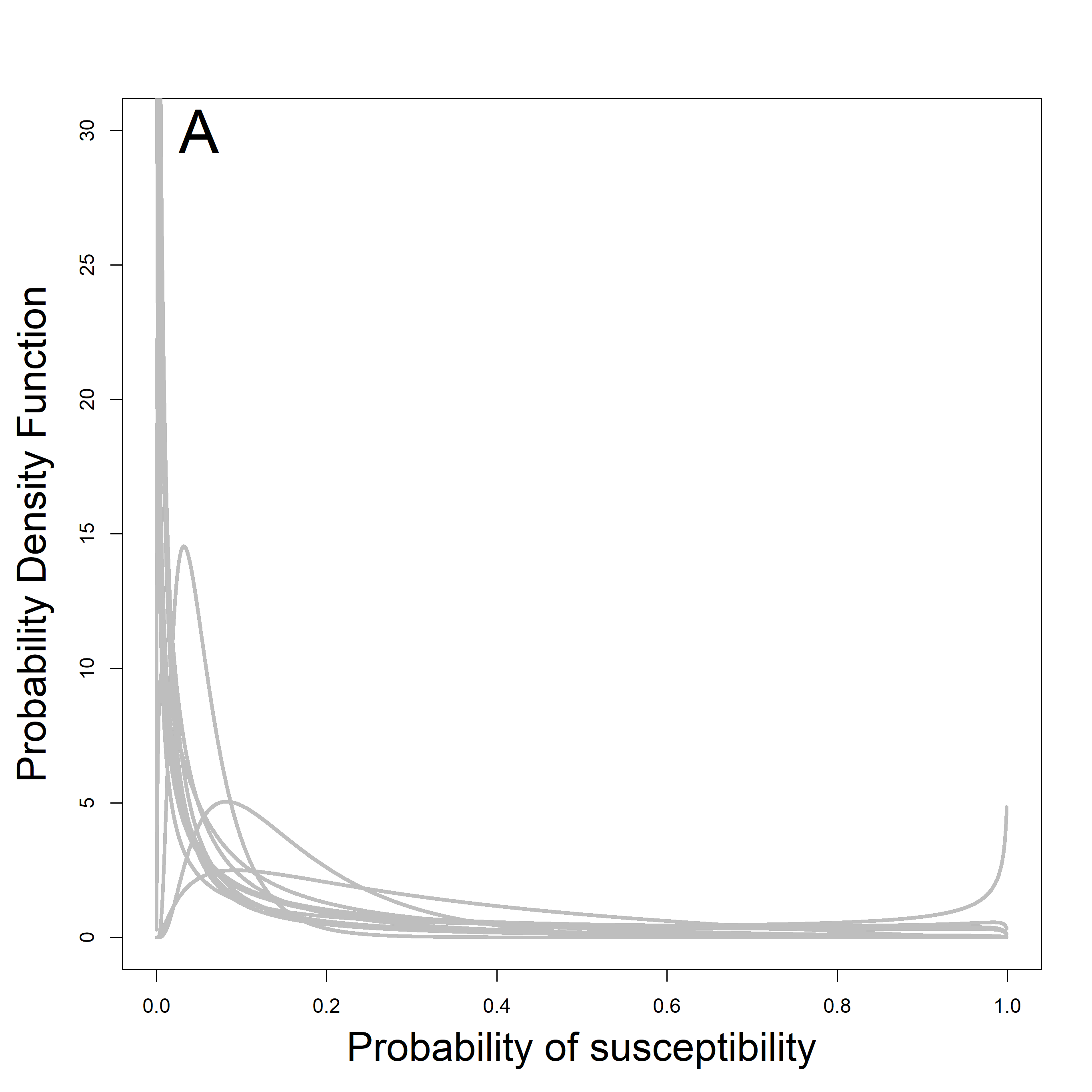

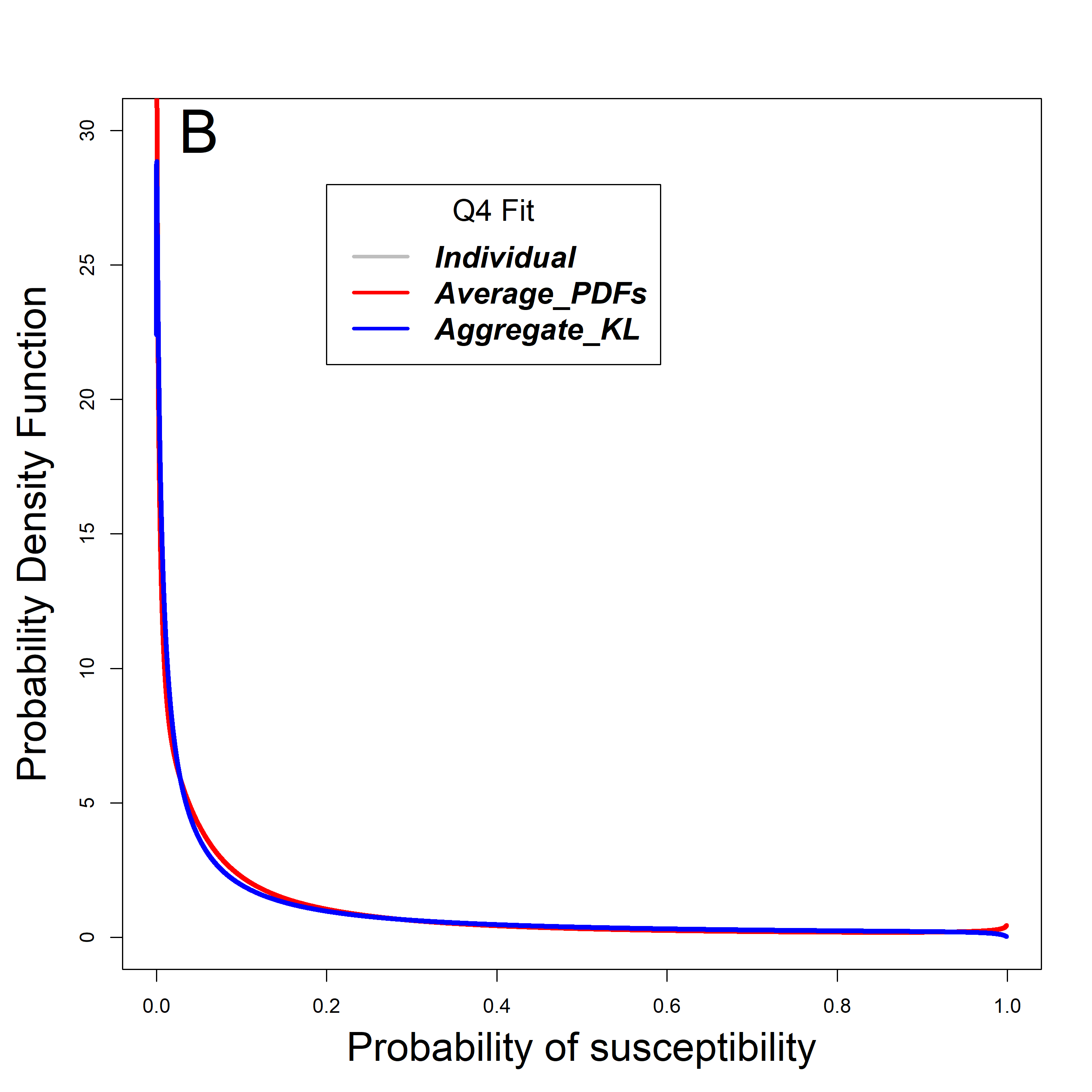


**Figure S6.** Expert panel responses to Question 5 — What is the ratio of the ID50 of an adult bat relative to that of an adult human? (A) fitted probability distributions (grey lines) for individual experts, and (B) aggregate fitted distributions across two groups of experts. Group 1 (red line) included four experts whose best guess for the bat:human ID50 ratio was ≤ 1 (i.e., the dose required for infection was less for bats than humans). Group 2 (pink line) included eight experts whose best guess for the bat:human ID50 ratio was > 1 (i.e., the dose required for infection was greater for bats than humans). The mixed distribution (blue line) for both groups has a median of 2.24 and an 80-percent confidence interval of (0.11, 9.88). Expert responses were later transformed into the relative susceptibility parameter, r, for direct use in the infection risk model.


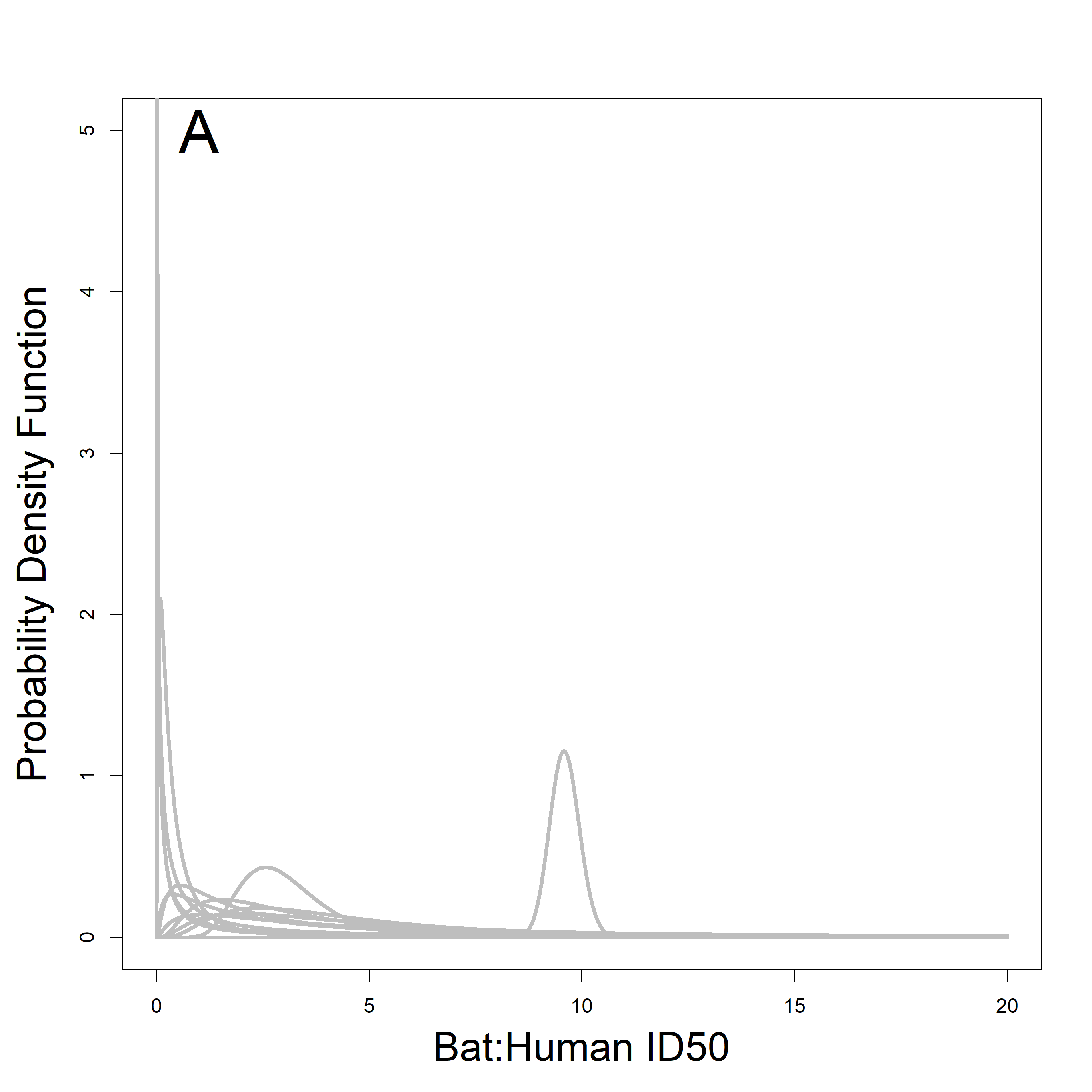

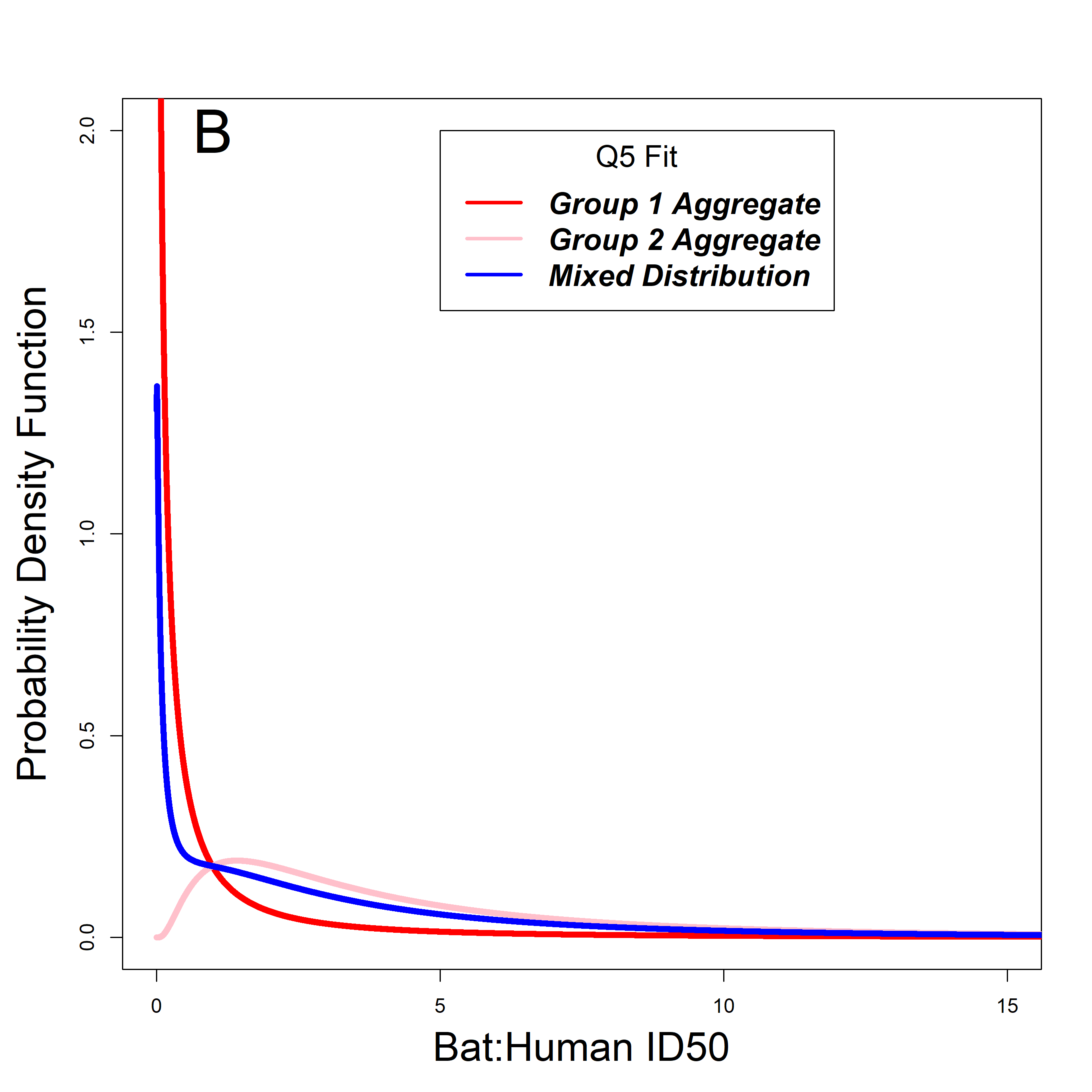


**Figure S7.** Expert panel responses to Question 6 — Of 100 little brown bats found to be infected with SARS-CoV-2 (at a time before bat-to-bat transmission could occur) in a hibernacula with WNS, how many would have been infected through the direct contact pathway rather than the aerosol pathway? (A) fitted probability distributions for individual experts (as fraction), and (B) average and fitted distributions across experts. The aggregate distribution has a median of 0.09 and an 80-percent confidence interval of (0.003 – 0.762).

**
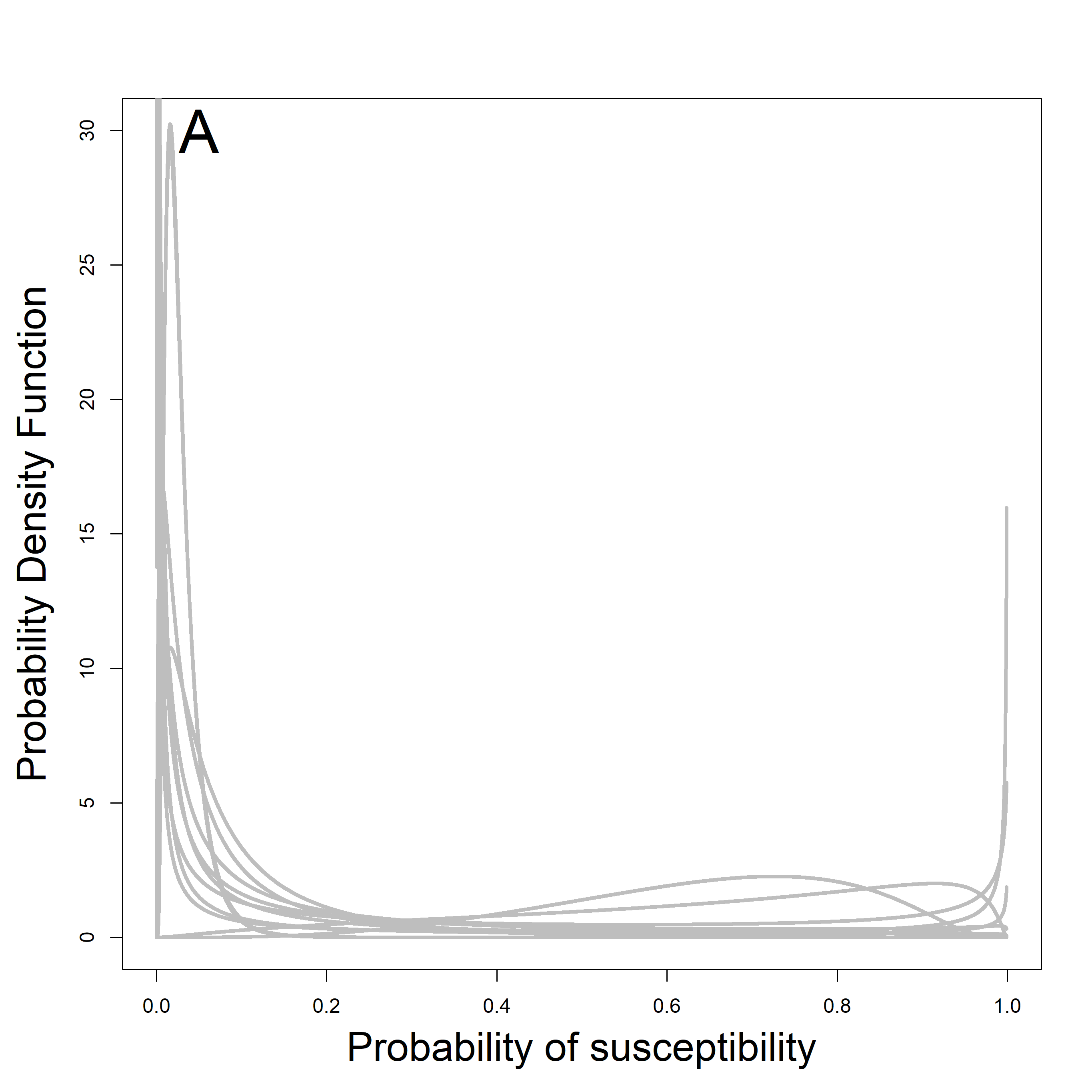

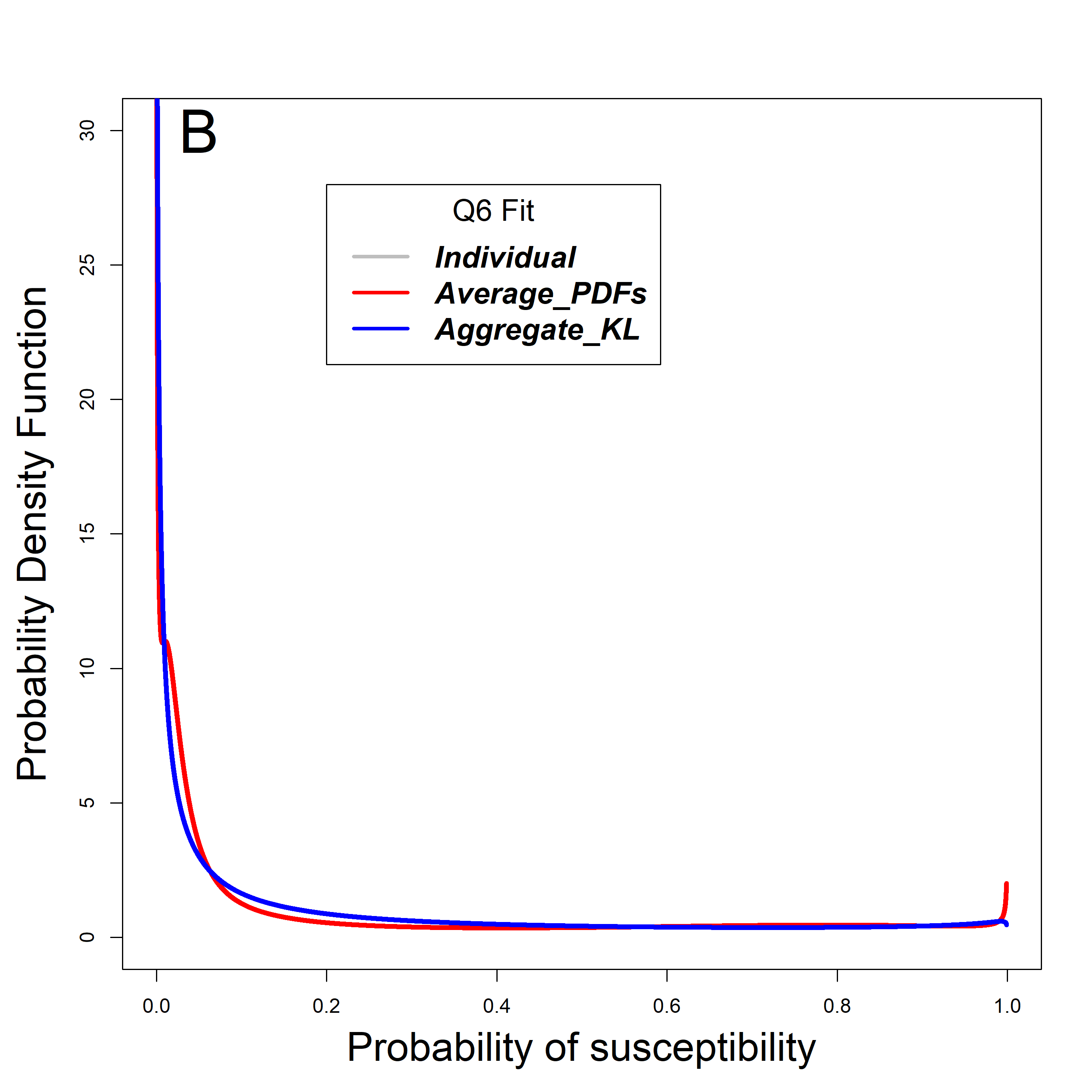
**

**Figure S8.** Expert panel responses to Question 7 — Of 100 big brown bats found to be infected with SARS-CoV-2 (at a time before bat-to-bat transmission could occur) in a hibernacula with WNS, how many would have been infected through the direct contact pathway rather than the aerosol pathway? (A) fitted probability distributions for individual experts (as fraction), and (B) average and fitted distributions across experts. The aggregate distribution has a median of 0.07 and an 80-percent confidence interval of (0.002, 0.721).


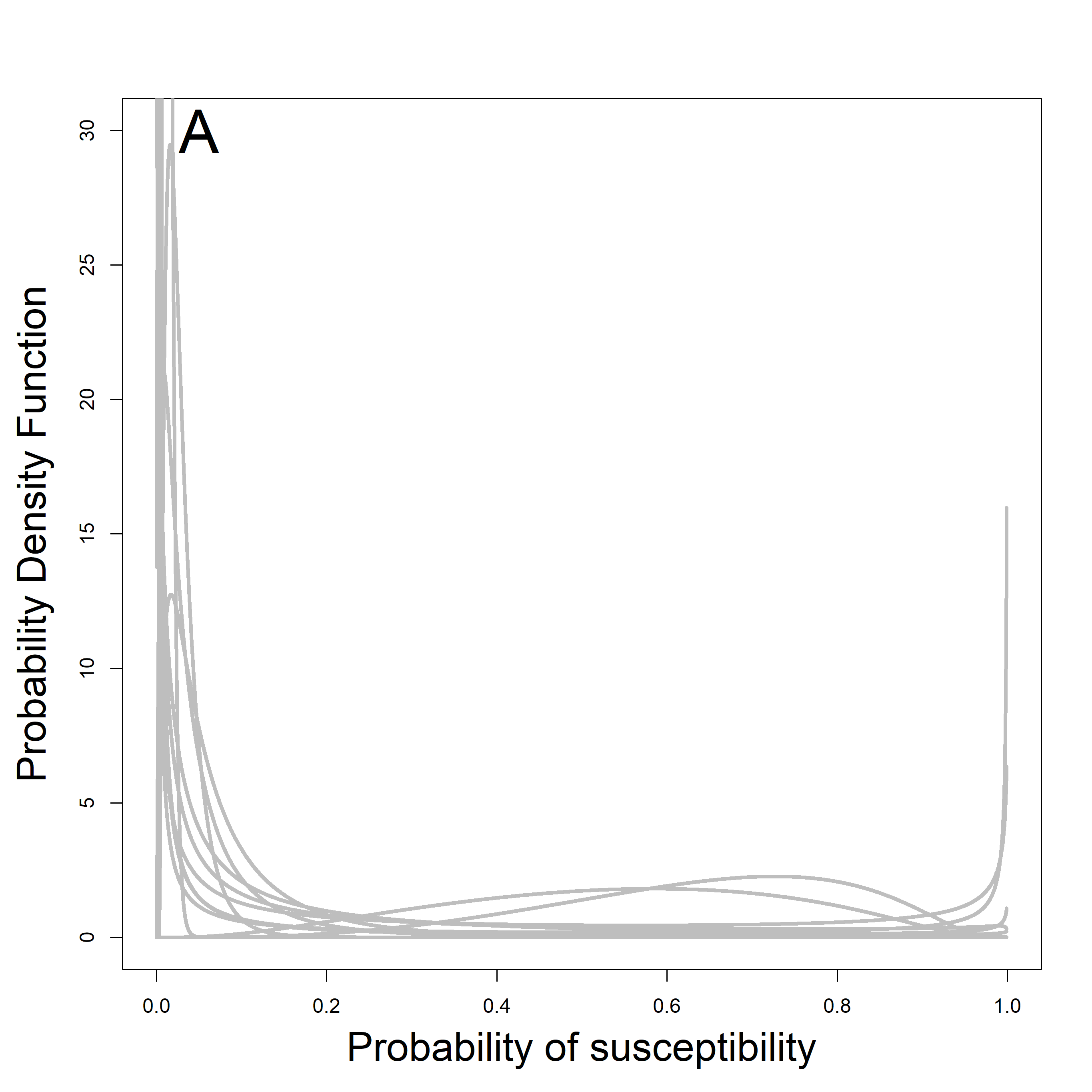

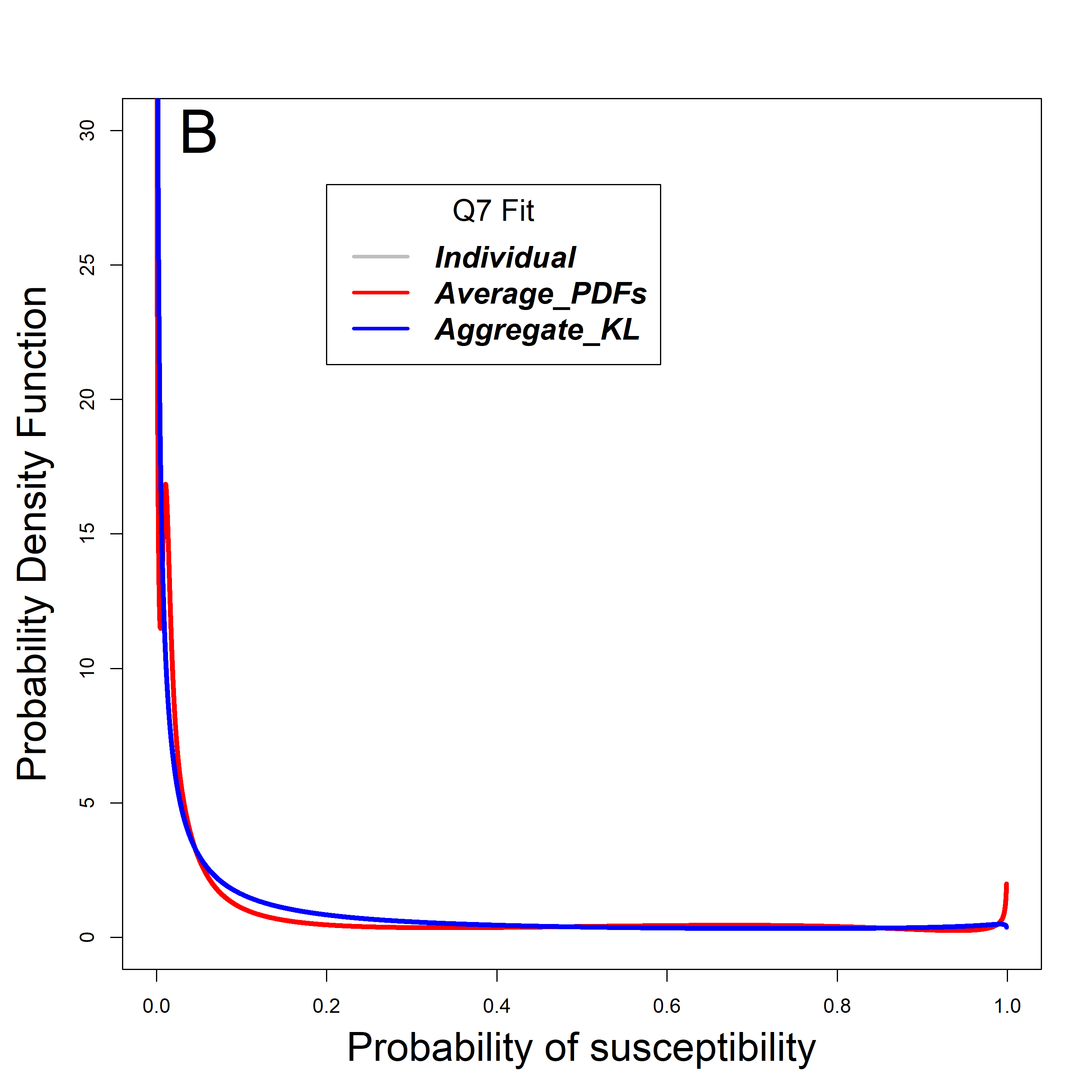


**Figure S9.** Expert panel responses to Question 8 — Of 100 little brown bats found to be infected with SARS-CoV-2 (at a time before bat-to-bat transmission could occur) in a hibernacula without WNS, how many would have been infected through the direct contact pathway rather than the aerosol pathway? (A) fitted probability distributions for individual experts (as fraction), and (B) average and fitted distributions across experts. The aggregate distribution has a median of 0.08 and an 80-percent confidence interval of (0.002, 0.824).


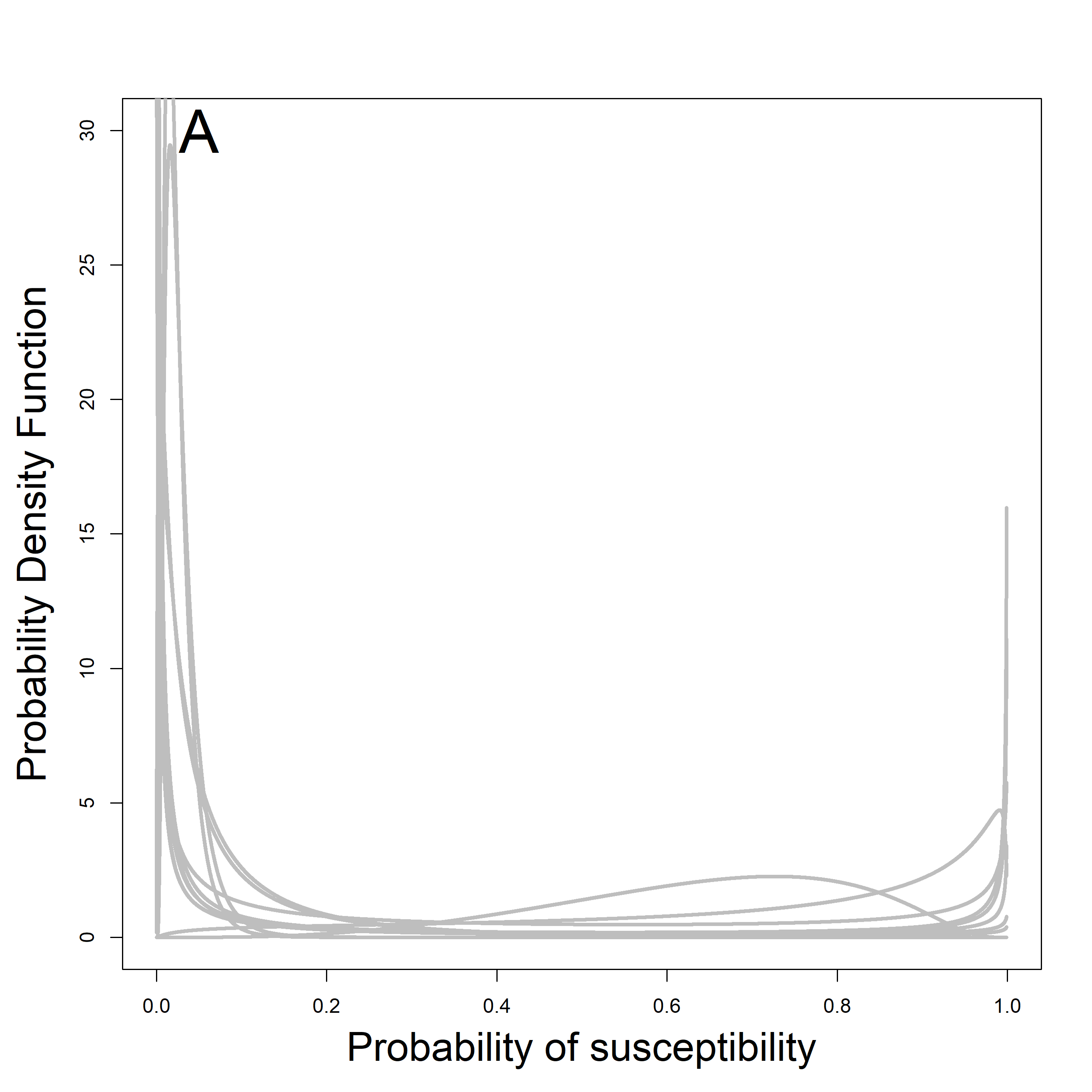

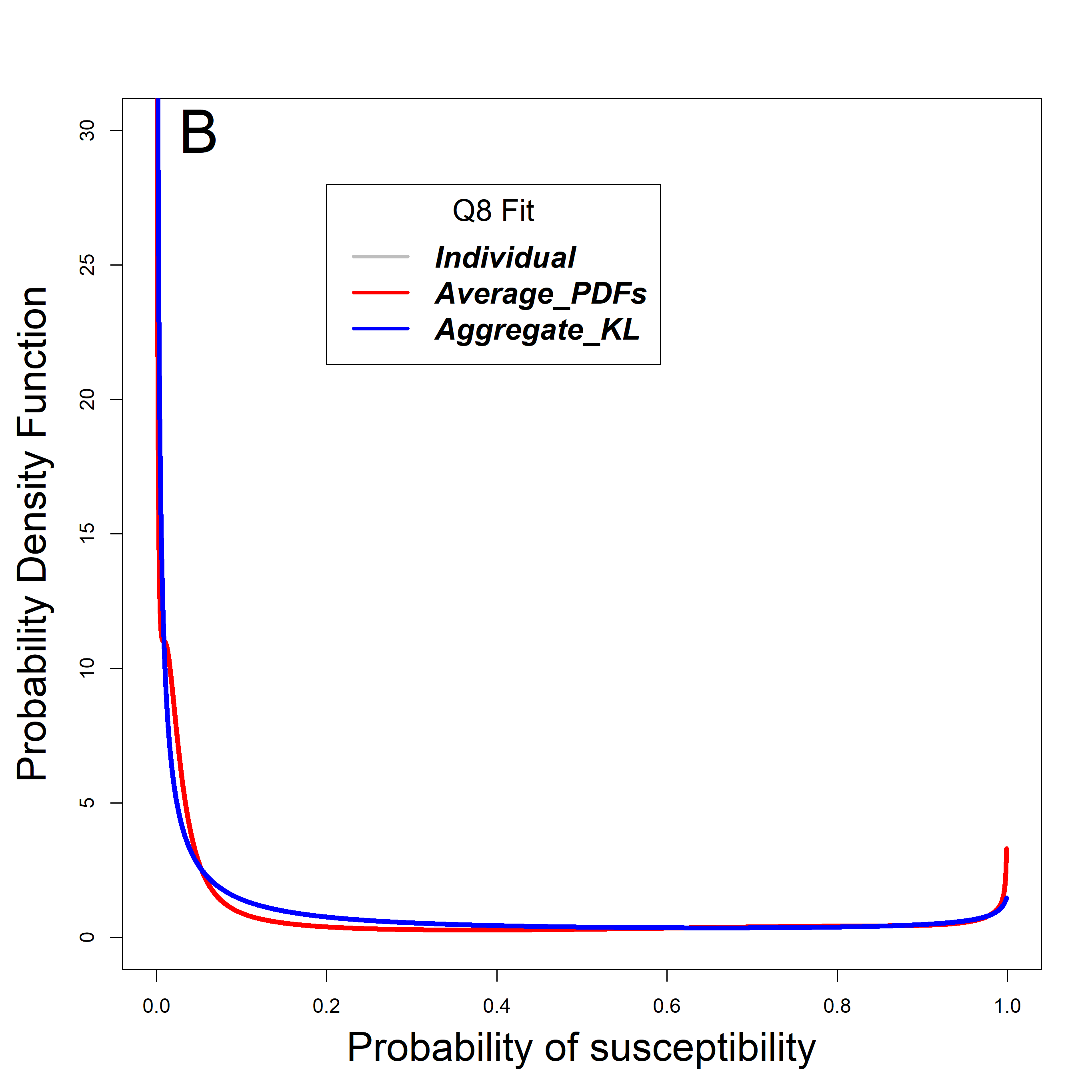


**Figure S10.** Expert panel responses to Question 9 — Of 100 big brown bats found to be infected with SARS-CoV-2 (at a time before bat-to-bat transmission could occur) in a hibernacula without WNS, how many would have been infected through the direct contact pathway rather than the aerosol pathway? (A) fitted probability distributions for individual experts (as fraction), and (B) average and fitted distributions across experts. The aggregate distribution has a median of 0.05 and an 80-percent confidence interval of (0.001, 0.694).


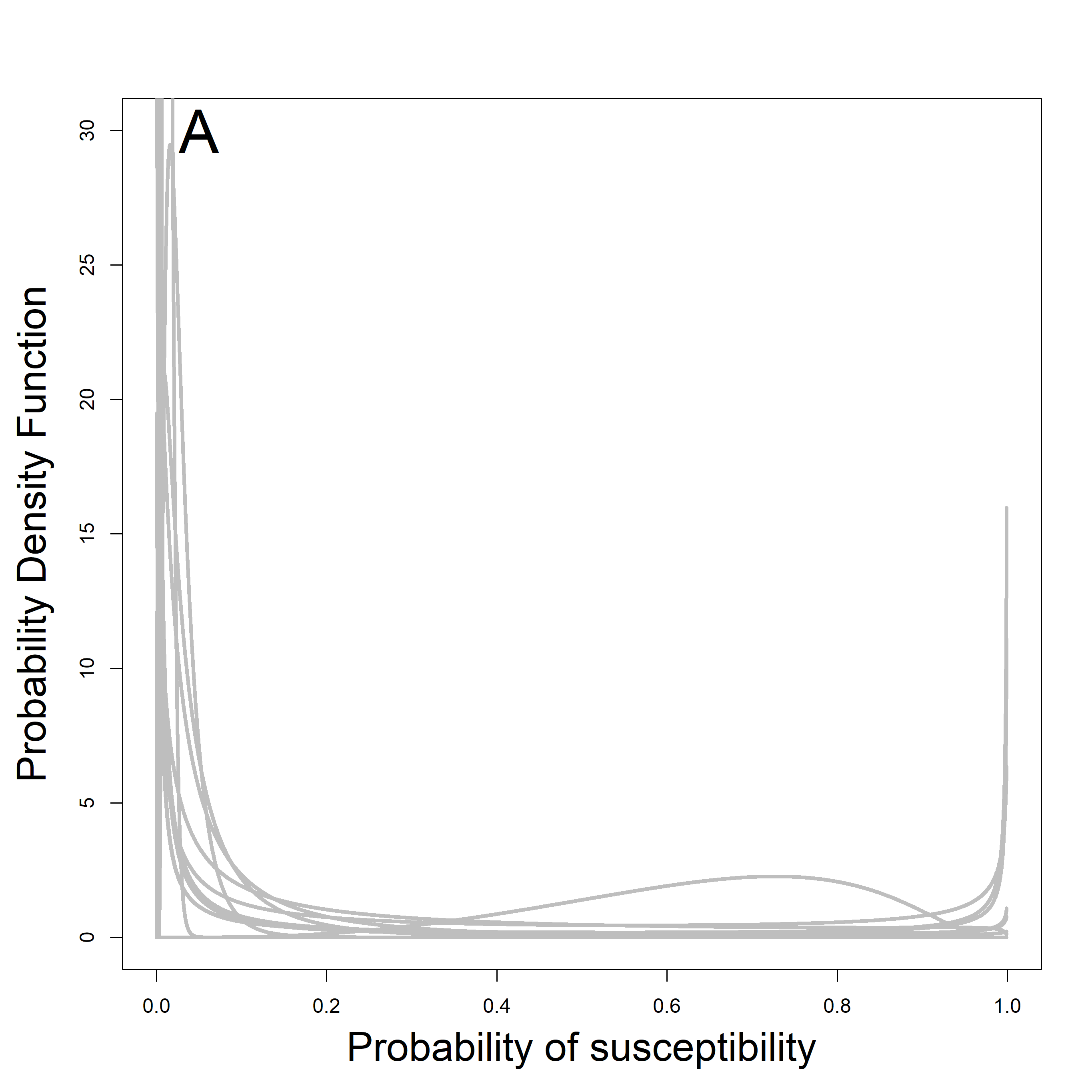

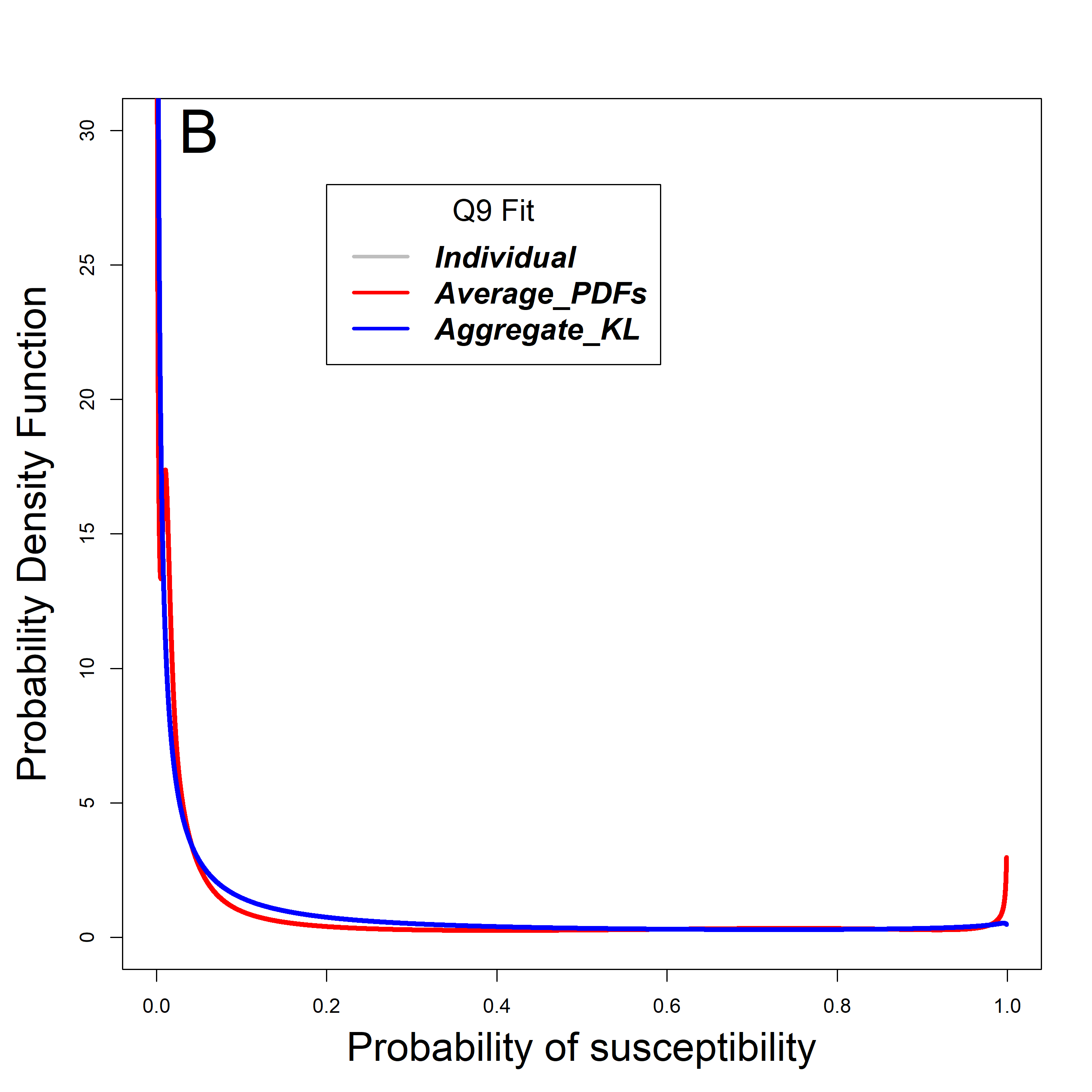


**Figure S11.** Expert panel responses to Question 10 — Of 100 Mexican free-tailed bats found to be infected with SARS-CoV-2 (at a time before bat-to-bat transmission could occur) in a hibernacula without WNS, how many would have been infected through the direct contact pathway rather than the aerosol pathway? (A) fitted probability distributions for individual experts (as fraction), and (B) average and fitted distributions across experts. The aggregate distribution has a median of 0.10 and an 80-percent confidence interval of (0.002, 0.864).


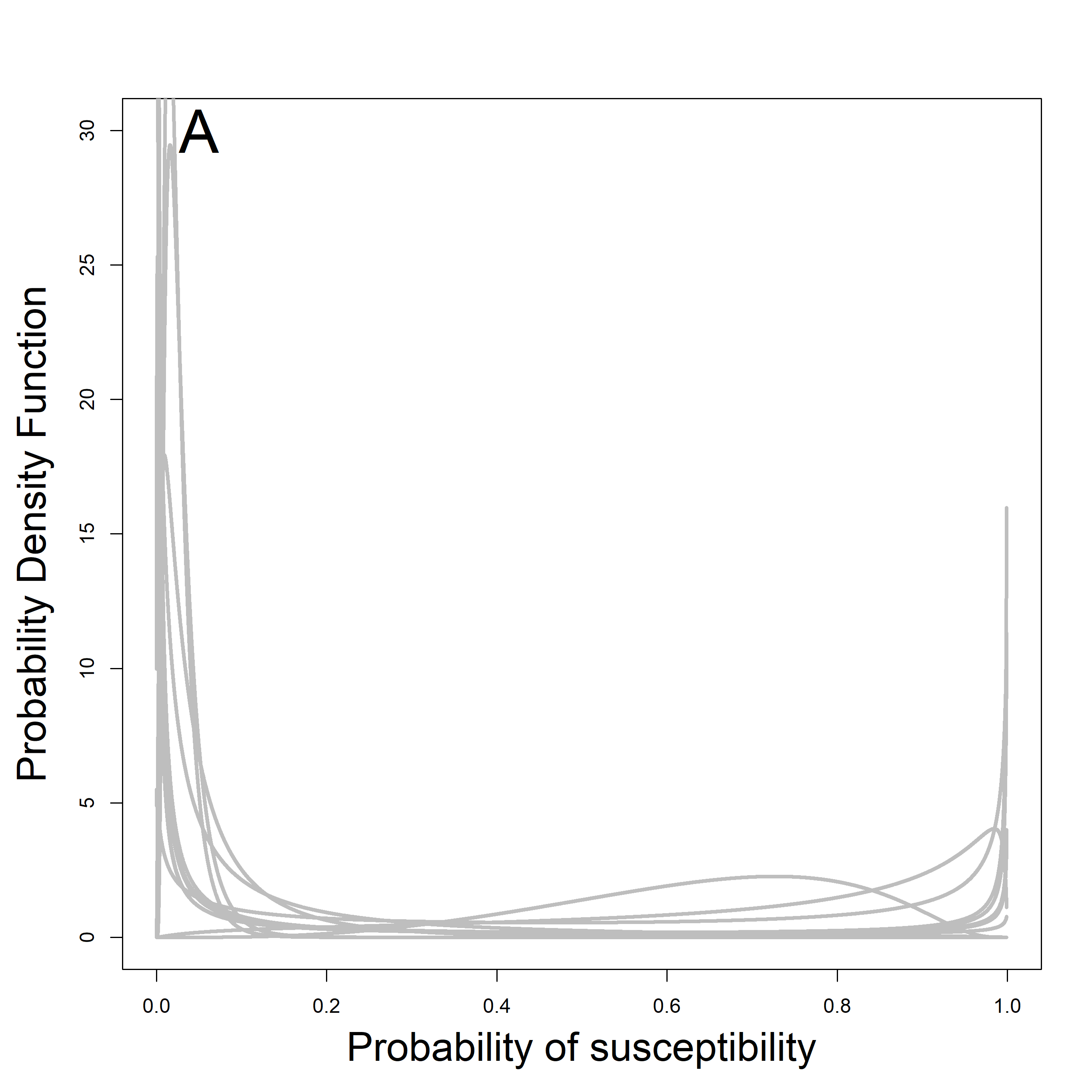

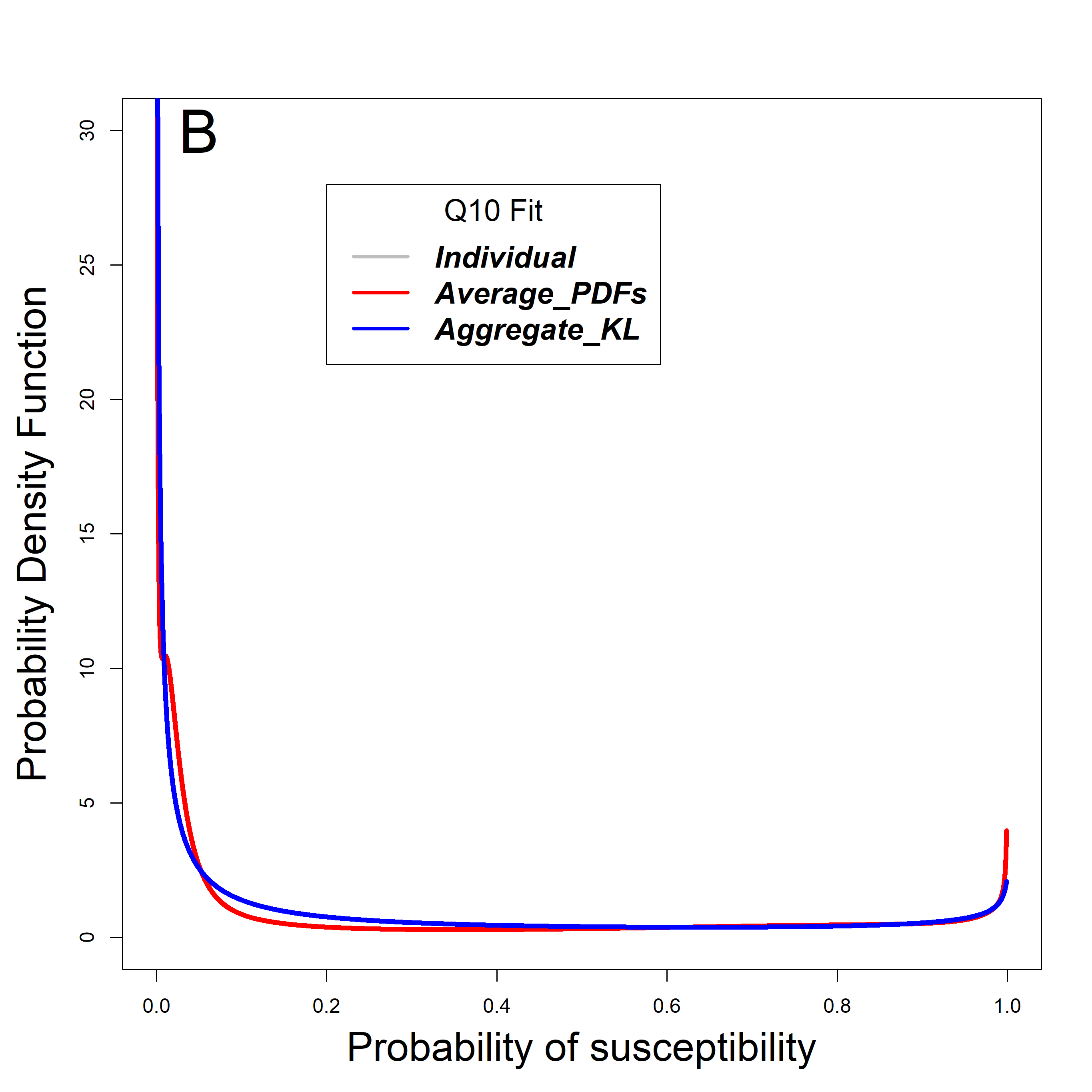

Supplement: Supplementary file 1 — Appendix S1: Supporting information [file CSP2-3-e410-s001.docx]
